# Genome-wide transcriptional responses of two metal-tolerant symbiotic *Mesorhizobium* isolates to Zinc and Cadmium exposure

**DOI:** 10.1186/1471-2164-14-292

**Published:** 2013-04-30

**Authors:** Géraldine Maynaud, Brigitte Brunel, Damien Mornico, Maxime Durot, Dany Severac, Emeric Dubois, Elisabeth Navarro, Jean-Claude Cleyet-Marel, Antoine Le Quéré

**Affiliations:** 1 INRA USC1242, Montpellier Cedex 5 F-34398, France; 2 Montpellier SupAgro, Montpellier 34000, France; 3 CEA, IG, Genoscope, 2 rue Gaston Crémieux, CP5706, Evry Cedex F-91057, France; 4 Montpellier GenomiX, c/o Institut de Génomique Fonctionnelle, 141 rue de la Cardonille, Montpellier Cedex 34 094, France; 5 IRD, Laboratoire des Symbioses Tropicales et Méditerranéennes UMR113, IRD/INRA/CIRAD/Montpellier SupAgro/Université Montpellier II, Montpellier F34000, France

**Keywords:** *Mesorhizobium*, Metal tolerant rhizobia, Zinc, Cadmium, Comparative genomics and transcriptomics, RNAseq, *znu*ABC

## Abstract

**Background:**

*Mesorhizobium metallidurans* STM 2683^T^ and *Mesorhizobium* sp. strain STM 4661 were isolated from nodules of the metallicolous legume *Anthyllis vulneraria* from distant mining spoils. They tolerate unusually high Zinc and Cadmium concentrations as compared to other mesorhizobia. This work aims to study the gene expression profiles associated with Zinc or Cadmium exposure and to identify genes involved in metal tolerance in these two metallicolous *Mesorhizobium* strains of interest for mine phytostabilization purposes.

**Results:**

The draft genomes of the two *Mezorhizobium* strains were sequenced and used to map RNAseq data obtained after Zinc or Cadmium stresses. Comparative genomics and transcriptomics allowed the rapid discovery of metal-specific or/and strain-specific genes. Respectively 1.05% (72/6,844) and 0.97% (68/6,994) predicted Coding DNA Sequences (CDS) for STM 2683 and STM 4661 were significantly differentially expressed upon metal exposure. Among these, a significant number of CDS involved in transport (13/72 and 13/68 for STM 2683 and STM 4661, respectively) and sequestration (15/72 and 16/68 for STM 2683 and STM 4661, respectively) were identified. Thirteen CDS presented homologs in both strains and were differentially regulated by Zinc and/or Cadmium. For instance, several P_IB_-type ATPases and genes likely to participate in metal sequestration were identified. Among the conserved CDS that showed differential regulation in the two isolates, we also found *znu*ABC homologs encoding for a high affinity ABC-type Zinc import system probably involved in Zinc homeostasis. Additionally, global analyses suggested that both metals also repressed significantly the translational machinery.

**Conclusions:**

The comparative RNAseq-based approach revealed a relatively low number of genes significantly regulated in the two *Mesorhizobium* strains. Very few of them were involved in the non-specific metal response, indicating that the approach was well suited for identifying genes that specifically respond to Zinc and Cadmium. Among significantly up-regulated genes, several encode metal efflux and sequestration systems which can be considered as the most widely represented mechanisms of rhizobial metal tolerance. Downstream functional studies will increase successful phytostabilization strategies by selecting appropriate metallicolous rhizobial partners.

## Background

Metal extraction activities generate large amounts of contaminated materials. Mining spoils are major sources of pollution as they disperse into the surrounding environment through aerial or water erosion and can be found several kilometers away from their original site of deposit. Such sources of pollution pose significant risks to public health [1] and to ecosystem dynamics due to reduced biodiversity resulting from low plant coverage caused by soil toxicity [2]. While remediation through chemical extraction destructs soils and is more expensive, phytoremediation strategies appear as more environment-friendly and constitute long term solutions to reduce metal toxicity in polluted sites. In highly polluted soils like mining soils, where the removal of metals by phytoextraction using hyperaccumulator plants is not efficient due to the slowness of the process, the best adapted method is phytostabilization [3]. This consists in limiting the dissemination of toxic metals by using metallicolous plants, *i.e.* metal tolerant plants, to establish a persistent plant cover and prevent pollution through erosion. Such a phytostabilization approach is nevertheless challenging since it requires metallicolous plants able to grow in soils where nutrients are most often dramatically scarce. Legume/rhizobia symbioses which transform atmospheric dinitrogen into organic nitrogen is of ecological interest here as it can improve natural soil fertility and thereby allow the colonization of other plant species and the installation of a plant cover [4,5]. However, survival and proliferation of organisms on metal contaminated sites depends on their capacity to tolerate high metal concentrations and requires the acquisition of resistance mechanisms.

A recent study conducted in a former mining area contaminated by Zinc (Zn), Cadmium (Cd) and Lead (Pb), named “les Malines Mining District” in the South of France, allowed the description of 116 plant species, some of which may be used in phytoremediation projects [6]. Among the plant species listed, a leguminous plant, *Anthyllis vulneraria,* is of particular interest as it can enter in symbiosis with rhizobia. A metal tolerant symbiotic partner of *A. vulneraria* was recently characterized as a new species of *Mesorhizobium* and named *M. metallidurans*[7]. Additionally, the *Anthyllis*/*Mesorhizobium* symbiosis has been identified in the mining site of Eylie in the Pyrenees (France). Eylie’s mine soil presents a geochemical background similar to the Avinières mine where *M. metallidurans* was found. It is highly polluted by Zn, Cd and Pb (14,300 mg kg^-1^, 23 mg kg^-1^ and 4,253 mg kg^-1^, respectively) but displays higher organic carbon content [5] than the Avinières soil. *Mesorhizobium* strains isolated from *Anthyllis* are (i) capable of entering in symbiosis with metal-tolerant ecotypes of *A. vulneraria* and (ii) tolerant to several metals including Zn and Cd ; therefore, they can be used in future phytostabilization strategies and constitute a good rhizobial model for studying the mechanisms they have evolved to tolerate high metal concentrations.

Prokaryotes have developed several complex molecular mechanisms to deal with ionic homeostasis or metal toxics [8-10]. The most and best studied metal resistant model bacterium is *Cupriavidus metallidurans* CH34, which harbors in its genome an exceptionally high number of metal resistance mechanisms [11]. The major mechanisms bacteria use to counteract metal toxicity consist in (i) permeability barriers, (ii) effluxes via P_IB_-type ATPases, Resistant Nodulation cell-Division proteins (RNDs), Cation Diffusion Facilitor proteins (CDFs) or Major Facilitor Superfamily systems (MFSs), (iii) intracellular and/or extracellular sequestration and (iv) transformation of metals into a less toxic form by enzymatic detoxification [8]. Efflux systems represent the largest category of metal-resistance systems [9]. In the model bacterium *C. metallidurans*, these mechanisms are in most cases not metal specific and under the control of a complex regulatory network involving several clusters of genes and functions [12].

In order to identify genes or functions in an organism of interest, genome sequences can be produced and analyzed using comparative genomics tools. Although genomic sequence data allow for the listing of putative gene contents in an organism, they do not provide any functional evidence. To go one step further, genome-wide transcriptional analyses allow for rapid identification of genes or loci that are transcriptionally active and associated with a particular physiological state. Such transcriptomic studies can now be available for any organism at a relatively low cost using high-throughput sequencing techniques in RNA sequencing (RNAseq) experiments.

In this paper, the main goals are to study gene expression profiles associated with Zn or Cd exposure and to identify genes involved in Cd and Zn tolerance in two symbiotic *Mesorhizobium* isolates using comparative genomics and transcriptomics. Two strains isolated from mining spoils of distinct geographical origins are compared: strain STM 2683 was isolated from the Avinières mine in the Cévennes area [7] and strain STM 4661 was isolated from the Eylie mine in the Pyrénées Ariégeoises (France). The draft genome sequences were produced using mate-pair 454 pyrosequencing and analyzed using an automated microbial genome annotation pipeline. RNAseq analyses were performed on bacteria exposed for half a generation time to mild Zn or Cd stresses which affect growth negatively but only to a low extent. Using mild exposure allows for the detection of genes and mechanisms specifically induced in the response to metals while avoiding all stresses related to complex responses induced when submitting bacteria to high growth-inhibiting or lethal metal concentrations.

## Results and discussion

### Effect of Zn and Cd on *Mesorhizobium metallidurans* STM 2683^T^ and *Mesorhizobium* sp. STM 4661 growth

**Figure 1:**
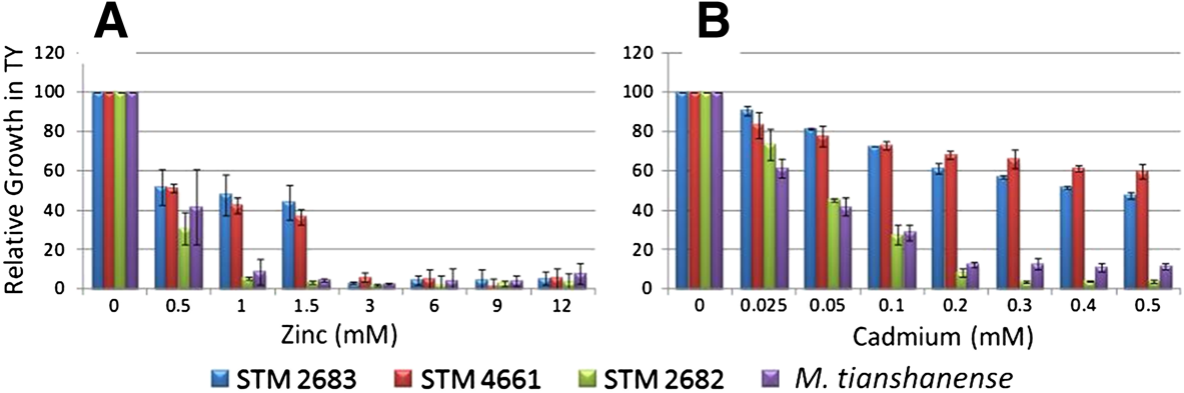
**Effect of Zinc and Cadmium on the growth of selected mesorhizobia.** Relative growth of *Mesorhizobium* strains STM 2683, STM 4661, STM 2682 and *M. tianshanense* in TY medium complemented with various concentrations of Zinc (**A**) or Cadmium (**B**). The results are the means of three replicates. Bars indicate the standard deviations from the means (±SD).

The effect of Zn or Cd addition to the TY growth medium was tested for the two metal-tolerant *Mesorhizobium* strains (STM 2683 and STM 4661) and for the metal-sensitive strains *M. tianshanense* (ORS 2740^T^) and *Mesorhizobium* sp. STM 2682 (Figure 1). Strains STM 2683 and STM 4661 were able to grow in media containing higher concentrations of Zn or Cd when compared to sensitive strains (MIC = 3 mM Zn and MIC > 0.5 mM Cd). In TY medium, the closely related *Mesorhizobium* strain STM 2682 or *M. tianshanense* were not able to grow when Zn or Cd concentrations were raised to 1 mM or 0.2 mM, respectively (Figure 1). Aiming at characterizing genes responding to Zn or Cd exposure in our mesorhizobial strains, three RNA libraries previously depleted in rRNA for both sequenced strains were prepared. The experimental design included exposure to Cd or Zn at concentrations (0.025 and 0.5 mM, respectively) that were high enough to affect mesorhizobia growth without being lethal (Figure 1). Such conditions were chosen in order to ascertain bio-availability of the metals and to avoid general and unspecific stress responses which would lead in the deregulation of a large number of genes that are not directly linked to the metal response. Furthermore, we used a short time exposure (approximately half a generation time) to these metals in order to detect genes and functions involved in the early response to the metals studied.

### Genome sequencing, assembly and automated annotation

In order to identify the genetic bases of our two metal-tolerant mesorhizobia and to use them as references to map sequence reads from the transcriptome data sets, the draft genomes of the two studied strains were first sequenced using half a 454 run (Titanium kit) with paired-end reads from an 8 kb genomic bank for each isolate. After the assembly, the comparison with *M. huakuii* MAFF 303099 genome (chromosome accession number: BA000012, plasmid pMLa: BA000013 and pMLb: AP003017) led for both genomes to the identification and organization of 1 chromosomal (~6 Mb) and 1 plasmidic (~250 Kb) scaffolds. These genomes were integrated into the MicroScope platform [13] to perform automatic and expert annotation of the genes, providing 6,908 genomic objects for STM 2683 and 7,065 for STM 4661. Genome sequences are also available from European Nucleotide Archive under accession numbers CAUM01000001-CAUM01000191 for strain STM 2683 and CAAF010000001-CAAF010000089 for strain STM 4661. The general genome characteristics for the two analyzed strains are listed in Table 1. As expected, CDS located on the plasmids were less conserved. We found that only 12% and 15% of CDS located on plasmids of STM 2683 and STM 4661 respectively possessed at least one homolog in the other strain. Despite the lower conservation observed for genes located on plasmids, we did not identify an over-representation of operons or genes that were associated with metals. Such results suggest that plasmids harbor accessory genes which are not specialized in metal adaptation in the strains STM 2683 and STM 4661 contrary to the model *C. metallidurans* CH34 where most metal-responsive genes are plasmid-borne [14].

**Table 1:** Genome overview after automated annotation in MAGE

**Characteristics**	**STM 2683**		**STM 4661**	
	**Chromosome**	**Plasmid**	**Chromosome**	**Plasmid**
**Sequence length (bases)**	6,020,204	233,929	6,304,314	251,782
**GC (%)**	62.53	60.53	62.44	60.53
**Number of Scaffolds**	3	2	1	1
**Number of Contigs**	171	20	83	6
**Nosferatu Repeated Regions (%)**	5.4	2.47	4.99	0
**Average CDS length (bp)**	825.98	755.96	845.13	838.18
**Average intergenic length (bp)**	134.86	162.18	139.62	160.14
**Protein coding density (%)**	88.01	86.25	88.01	87.12
**Number of Genomic Objects (CDS, fCDS, rRNA, tRNA, miscRNA)**	6,628	280	6,791	274
**Number of CDSs**	6,512	272	6,683	267
**Number of fCDSs**	54	6	40	4
**Number of misc_RNAs**	14	2	16	3
**Number of rRNAs**	3	0	3	0
**Number of tRNAs**	45	0	49	0

In order to estimate genomic divergence between the two strains, we explored the proportions of homologous CDS as a function of the identity percentage. Homologous genes present in the two strains were retrieved with the comparative genomics phyloprofile tool in MicroScope. Using an alignment threshold of 80% identity over at least 80% of the query sequence and target size, approximately 4,200 homologous genes, present in both strains, were identified (4,183 CDS from STM 2683 presented at least one homolog in STM 4661 and 4,186 CDS from STM 4661 presented a minimum of one homolog in STM 2683). These results show that approximately two thirds of the genomes are conserved in the two sequenced metal-tolerant strains (data not shown).

A comparative genomic study was performed with the seven available sequenced *Mesorhizobium* genomes; three metallicolous genomes (*M. metallidurans* STM 2683^T^, *Mesorhizobium* sp. STM 4661 and *M. amorphae* CCNWGS0123) and four non-metallicolous genomes (*M. ciceri* bv. *biserrulae* WSM 1271, *M. opportunistum* WSM 2075, *M. huakuii* MAFF303099, *M. australicum* WSM 2073) : it shows that among annotated CDS, putative genes that encode transport and sequestration proteins were over-represented in the three metallicolous mesorhizobial genomes (data not shown).

### RNAseq analysis, mapping and statistical analyses using DESeq

The universal bacterial rRNA subtraction kit (Microbe Express, Ambion) allowed for the removal of most rRNAs. Qualitative analyses of RNA samples prior and after depletion indicated that total RNA was of high quality (RIN values ranging from 7 to 10) and that most 16S and 23S rRNA peaks were removed after depletion (Additional file 1). The mRNA samples were sequenced in 36 bp-cycles using the illumina HiSeq2000 (San diego, CA) with SBS technology. A lane of a FlowCell was used per sample. Image analyses and basecalling were conducted using the HiSeq Control Software (HCS 1.1.37.8) and RTA component (RTA 1.7.48). The RNAseq data are available from NCBI GEO datasets under the accession number GSE45693. The number of reads which passed the initial quality filter varied from 68.8 to ca 81.6 million and the quality analyses of the sequencing runs showed that the sequencing step was successful (data not shown). The general characteristics of the RNAseq data, listed in Table 2, showed homogeneity between treatments and strains. Notably, we found that more than 97% of the sequence reads were mapped at least once onto genomic objects identified via the automated annotation Microscope pipeline. If we consider that total bacterial RNA is composed of 95% rRNA, we found that the percentage of rRNA depletion was high (ranging from 92 to 96%) confirming that the kit used was well suited for our *Mesorhizobium* strains.

**Table 2:** RNAseq overview and mapping

Characteristics*	**STM 2683**			**STM 4661**		
	**Control (TY)**	**Zinc (0.5 mM)**	**Cadmium (0.025 mM)**	**Control (TY)**	**Zinc (0.5 mM)**	**Cadmium (0.025 mM)**
**Total read number**	81,308,886 (100%)	73,773,403 (100%)	68,813,483 (100%)	72,111,015 (100%)	74,765,870 (100%)	81,587,705 (100%)
**Nb of unmapped reads**	1,949,038 (2.40%)	2,031,313 (2.75%)	1,892,551 (2.75%)	1,623,268 (2.25%)	1,718,059 (2.30%)	1,776,552 (2.18%)
**Nb of reads mapped at least once**	79,359,848 (97.60%)	71,742,090 97.25%)	66,920,932 (97.25%)	70,487,747 (97.75%)	73,047,811 (97.70%)	79,811,153 (97.82%)
**Nb of reads mapped on rRNA**	41,428,635 (50.95%)	30,531,652 (41.39%)	32,224,949 (46.83%)	39,466,389 (54.73%)	37,982,860 (50.80%)	47,500,946 (58.22%)
rRNA depletion rate **	94.3	96.1	95.1	93.3	94.3	92.3
**Nb of reads kept (only reads uniquely mapped)**	78,729,668 (96.83%)	71,178,074 (96.48%)	66,380,750 (96.46%)	70,090,461 (97.20%)	72,683,423 (97.21%)	79,431,748 (97.36%)
**Nb of reads kept against chromosome**	78,147,818 (96.11%)	70,490,263 (95.55%)	65,861,607 (95.71%)	69,802,581 (96.80%)	72,348,246 (96.77%)	79,118,286 (96.97%)
**Nb of reads kept against plasmid**	581,850 (0.72%)	687,811 (0.93%)	519,143 (0.75%)	287,880 (0.40%)	335,177 (0.45%)	313,462 (0.38%)
Nb of reads kept for downstream analyses***	37,301,033 (45.86%)	40,646,422 (55.10%)	34,155,801 (49.64%)	30,624,072 (42.47%)	34,700,563 (46.41%)	31,930,802 (39.14%)

* Numbers of reads per sample obtained after mapping analyses are indicated together with their proportions relative to the total read numbers in parentheses.

** Estimated rRNA substitution rate, considering that 95% of the RNA was initially composed of rRNA.

***Only reads that uniquely mapped, and did not map rRNAs.

After removal of all rRNA reads, more than 30 million sequence reads for each treatment mapped uniquely onto genomic objects and were used to estimate gene expression. Interestingly, sequence reads were successfully assigned to all predicted genomic objects, suggesting that all CDS were transcribed and that sequencing was deep enough to cover the full transcriptomes. All read mapping data for STM 2683 and STM 4661 are accessible from the Microscope interface [15]. Using the MicroScope automated RNASeq pipeline, mapping results were then translated into raw read counts for each gene and processed through the DESeq statistical package [16] to normalize and test for differential expression between conditions (see Methods). Basically, DESeq estimates library size factors to normalize gene read counts between samples by assuming that a majority of genes have comparable expression levels in all samples. It then estimates gene expression dispersion due to biological and technical variations within each condition, and models it using negative binomial distributions. Genes for which expression levels significantly differ from the estimated dispersion are then called as differently expressed, and DESeq provides *p-values* adjusted for multiple testing with the Benjamini-Hochberg procedure [17] to control the false discovery rate (FDR). Although each sample (composed of 6 pooled biological replicates) was sequenced once, DESeq was able to estimate gene expression dispersion by assuming that most genes have similar expression across treatments. This method overestimates dispersion relatively to when sample replicates are available, resulting in more conservative differential expression calls and slightly lower sensitivity [16]. The data produced using DESeq for all predicted CDS are accessible from the Microscope interface [15].

**Figure 2:**
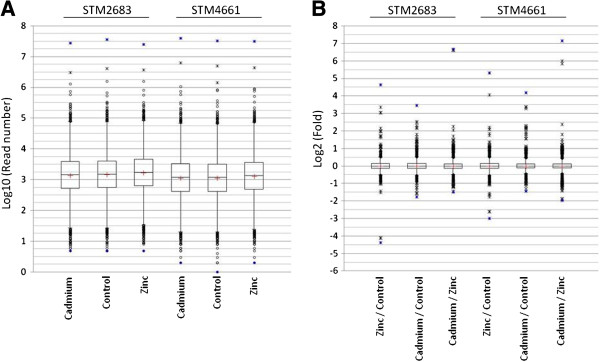
**Distributions of the RNAseq datasets using Box plot representations.** (**A**) Box plots representing expression level distributions (log10 of read numbers) for all CDS in the three treatments (Cadmium, Control or Zinc, bottom of the graph) and for the two isolates (STM 2683 and STM 4661, top of the graph). (**B**) Box plots representing the distributions of the log2 fold changes for all possible comparisons (bottom of the graph) for all CDS and for the two isolates (top of the graph). The mild and extreme outliers are represented by empty circles and stars, respectively, and whiskers correspond to + and – 1.5 of the Interquartile values (bars). Extreme values are in blue and mean values are represented by red crosses.

Possible ways to estimate the quality and reproducibility of the RNAseq data produced consist in comparing the distributions of (i) the read number per CDS prior to normalization and (ii) the differential expressions of CDS in all comparisons after normalization. Because we observed a wide magnitude in the read count numbers per CDS, a logarithmic transformation of these data was performed. A logarithmic base-2 transformation of fold changes was used to represent expression differences. The Additional file 2 reports the descriptive statistics obtained from our quantitative RNAseq datasets. The variances obtained in expression statistical analyses (Additional file 2) were low (ranging from 0.058 to 0.075), indicating that most predicted genes presented similar expression levels across treatments. Box plots can be used to compare the general characteristics of large datasets. These were produced in order to compare the distributions of our RNAseq data between treatments but also between strains (Figure 2). The median read number per CDS was above 1,000 (log value > 3). It varied from 1,432 to 1,714 for STM 2683 and from 1,180 to 1,342 for STM 4661 (Additional file 2). The lower median read number per CDS obtained from the RNAseq data of STM 4661 can be attributed to the larger number of reads mapped on ribosomal RNAs as well as the higher CDS number that was predicted in this isolate. Nevertheless, despite this shift in the median values, comparable distributions were found as indicated by box sizes and box plot whiskers (Figure 2A). The box plots representing the distributions of expression differences (corresponding to log2 fold changes) for all possible comparisons and for the two isolates were also very similar (Figure 2B). In this case, the interquartile values obtained were lower than 0.3 for all comparisons, which is equivalent to a negative or a positive fold change of 1.11 or less for 50% of the annotated CDS. Furthermore, whiskers sizes (+/− 1.5 of the interquartile values representing more than 95% of the data in our distributions) were similar between comparisons and isolates and close to a log2 fold change of +/− 0.5, showing that for more than 95% of the predicted CDS, fold changes under 1.41 were observed. These results indicate that the treatments did not have a profound effect at the genome-wide expression level and that the majority of CDS were not affected by the treatments. Histograms showing the distributions of the log10 read counts for each treatment and the log2 fold changes for all comparisons are presented in Additional files 3 and 4, respectively. Interestingly, they were very similar for all treatments and therefore were not strain-specific showing that the transcriptome sequencing step was reproducible at the quantitative level.

The box plot and histogram graphical representations showed that the distributions of the data were quantitatively comparable between treatments and strains. However, these representations do not allow to test if read numbers affect differential expression variance, especially for genes that are lowly transcribed. In order to visualize and estimate data dispersion according to read count levels, scatter plots and MA plots were produced for each Metal *vs* Control comparison (Additional files 5 and 6). These representations show that the data were highly centered in all comparisons. Correlation factors (R^2^ values) were calculated for CDS classified by their mean read numbers for all scatter plots (Additional file 5). As expected, the correlation factor generally increased with the read count number per CDS, even though a rather high correlation factor (R^2^ > 0.73) for CDSs with a mean read number under 100 was found. Altogether, our data show that the RNAseq approach produced high quality and quantitative data, even for lowly expressed CDS.

### Quantitative PCR on selected genes

The reliability of our RNAseq data can be assessed by comparing the differential expression of a set of regulated genes using an alternative approach such as quantitative real time PCR (qPCR). The relative expression of five genes present in both studied microbial genomes was estimated using qPCR. Three genes presenting significant differential expression values after DESeq analyses in our RNAseq data (genes encoding for a metal-translocating P_IB_-type ATPase: MESS2v1_740030 / MESS4v1_360013, a periplasmic binding protein of the ABC-type transporter: MESS2v1_300037 / MESS4v1_520016, and a putative Signal peptidase II: MESS2v1_740019 / MESS4v1_360023 in STM 2683 / STM 4661, respectively) were chosen and analyzed using two reference genes (*rec*A *and gln*A) whose expression was unaltered by metal treatment. The logarithm (base 2) ratios (Zn or Cd treatment / control treatment) were assessed in the two strains using the same pooled extracted RNA samples that were used for the RNAseq. The relative expression values obtained by qPCR for each gene and for the two treatments were calculated using the mean values obtained using the two reference gene expression values as standards. These relative expression levels were compared to those obtained with the RNAseq approach (Additional file 7). We found that the genes identified as significantly regulated using the genome-wide approach were also differentially expressed by qPCR for both isolates (Additional file 7A, B, D and E). Despite a higher up-regulation observed by qPCR for MESS2v1_740019 upon Cd treatment on STM 2683, a correlation factor (R^2^) above 0.9 was found for both isolates when plotting the log2 (Metal / control) values obtained for the five selected genes using the genome-wide RNAseq approach against the qPCR data (Additional file 7C and F). Differences in the amplitude of the deregulation between the two techniques could be attributed to the PCR step used to amplify libraries prior to Illumina sequencing or to differences resulting from the mRNA purification procedure. Nevertheless, the high correlation between the two approaches shows that RNAseq data can be used to assess the global gene expression levels in our *Mesorhizobium* strains and more particularly to identify the genes specifically induced or repressed by Zn or Cd.

To estimate the biological variance of our data, the relative expression ratios of the same target genes were also studied by qPCR in STM 2683, using total RNA isolated from independent biological replicates as starting materials, and the ratios were compared to the results obtained using pooled RNA samples (Additional file 8). Again, a high correlation factor (above 0.99) was obtained between replicates (data not shown). We found that the relative expression values obtained from the pooled RNA samples and the biological replicates were very similar with both metal treatments and the standard deviations between technical replicates were comparable to those obtained in biological replicates (Additional file 8A and B) and were highly correlated (R^2^ > 0.98) (Additional file 8C). Altogether, the high correlation factors we obtained show that the RNAseq data produced in the present study contain reliable relative expression values. This is in agreement and in line with the RNAseq data analyses which showed high correlations of the read count numbers between treatments at the genome-wide level. Despite its relatively high cost, the RNAseq approach, which is based on transcript sequencing, represents a choice method for quantitative transcript measurements: the generated sequences are proofs of the corresponding gene transcriptional activities as compared to other indirect global approaches based on hybridization or qPCR which allow for the quantification of transcript levels for a limited set of targets.

### Functional composition of *Mesorhizobium* transcriptomes and alteration upon metal exposure

The functional COG classifications of all CDS in the two *Mesorhizobium* genomes under study were obtained automatically using COGNiTOR [18] (Table 3).

**Table 3:** COG functional assignment of CDS detected in the genomes of STM 2683 and STM 4661

**COG Class ID**	**Process**	**Description**	**CDS Number**	
			2683*	4661**
**B**	INFORMATION STORAGE AND PROCESSING	Chromatin structure and dynamics	5	6
**C**^**1, 2**^	METABOLISM	Energy production and conversion	370	393
**D**^**2**^	CELLULAR PROCESSES AND SIGNALING	Cell cycle control, cell division, chromosome partitioning	54	51
**E**^**1**^	METABOLISM	Amino acid transport and metabolism	1111	1135
**F**	METABOLISM	Nucleotide transport and metabolism	134	128
**G**^**1**^	METABOLISM	Carbohydrate transport and metabolism	628	604
**H**	METABOLISM	Coenzyme transport and metabolism	194	210
**I**	METABOLISM	Lipid transport and metabolism	279	295
**J**^**1, 2**^	INFORMATION STORAGE AND PROCESSING	Translation, ribosomal structure and biogenesis	247	257
**K**	INFORMATION STORAGE AND PROCESSING	Transcription	526	540
**L**^**2**^	INFORMATION STORAGE AND PROCESSING	Replication, recombination and repair	278	257
**M**^**1, 2**^	CELLULAR PROCESSES AND SIGNALING	Cell wall/membrane/envelope biogenesis	322	343
**N**^**2**^	CELLULAR PROCESSES AND SIGNALING	Cell motility	69	48
**O**^**2, 3**^	CELLULAR PROCESSES AND SIGNALING	Posttranslational modification, protein turnover, chaperones	227	213
**P**^**1, 3**^	METABOLISM	Inorganic ion transport and metabolism	629	654
**Q**	METABOLISM	Secondary metabolite biosynthesis, transport and catabolism	249	263
**R**^**1**^	POORLY CHARACTERIZED	General function prediction only	994	1058
**S**	POORLY CHARACTERIZED	Function unknown	421	454
**T**^**2**^	CELLULAR PROCESSES AND SIGNALING	Signal transduction mechanisms	237	237
**U**^**2**^	CELLULAR PROCESSES AND SIGNALING	Intracellular trafficking, secretion, and vesicular transport	107	91
**V**	CELLULAR PROCESSES AND SIGNALING	Defense mechanisms	204	205
**W**	CELLULAR PROCESSES AND SIGNALING	Extracellular structures	1	1

* 4,903 CDS out of 6,844 (71.6%) were assigned to at least one COG class ID for STM 2683.

** 5,042 CDS out of 6,994 (72.1%) were assigned to at least one COG Class ID for STM 4661.

**Figure 3:**
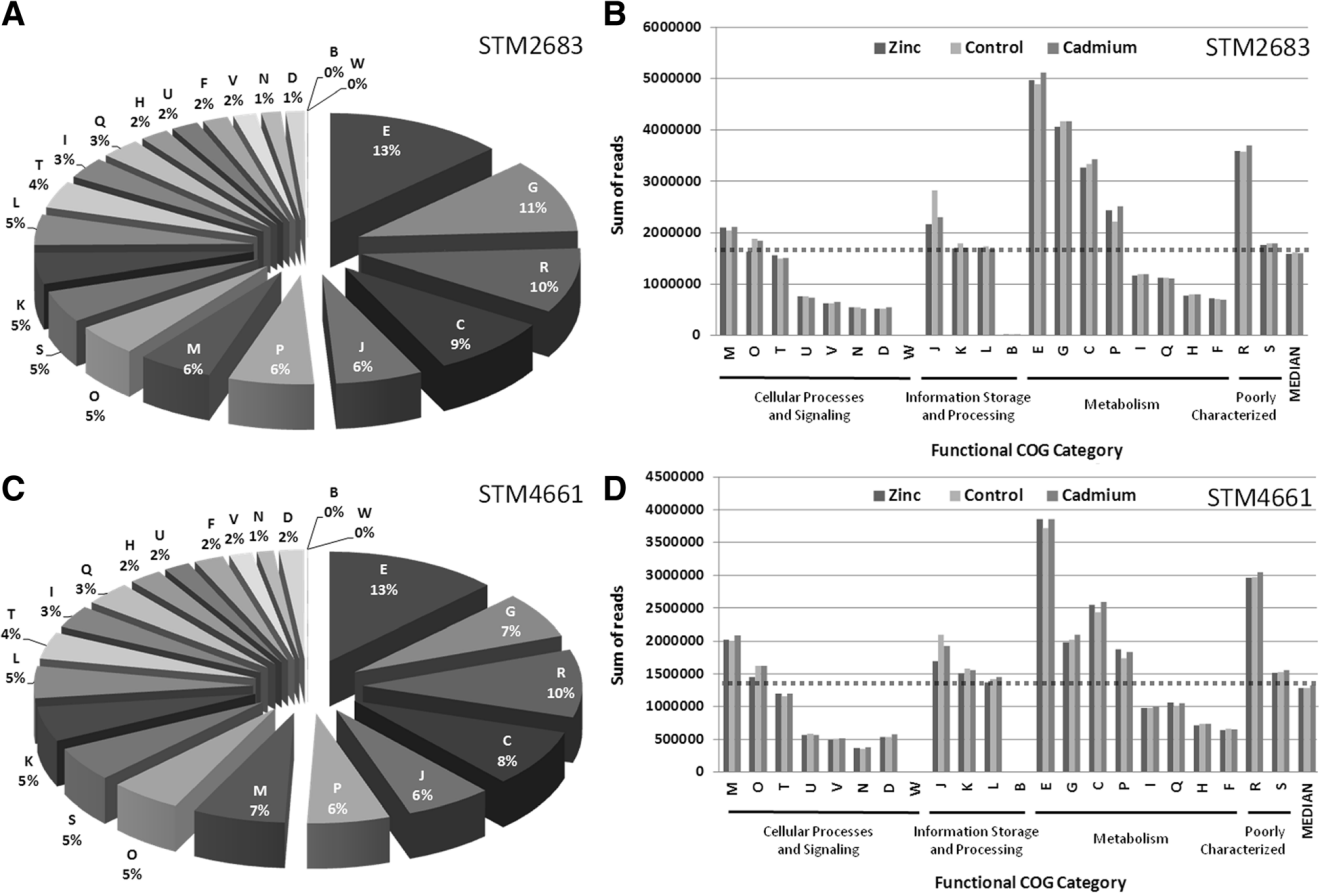
**Functional composition of the transcriptomes.** The proportions and absolute amounts of reads for CDS after COG class assignment are shown for strains STM 2683 (**A** and **B**) and STM 4661 (**C** and **D**). Chart pies (**A** and **C**) show the relative quantities of transcripts belonging to defined COG functional classes (B-W; see Table 3 for description) in the *Mesorhizobium* transcriptomes. Histograms representing the quantitative composition of the transcriptomes (B and D) organized by the biological Process and Class ID (bottom of histograms) show the total amounts of reads (indicative of transcript abundance) in each class and for each treatment (Zinc, Control or Cadmium). The median read numbers per class and per treatment are indicated as the last bars on the histograms which were used to draw the dotted line above which the defined classes are the most represented for both organisms and for all treatments (these class IDs and their proportions are indicated in white on the pie charts).

^1^ COG Class representing more than 5% of the transcriptomes for both strains and for all treatments (cf. Figure 3).

**Figure 4:**
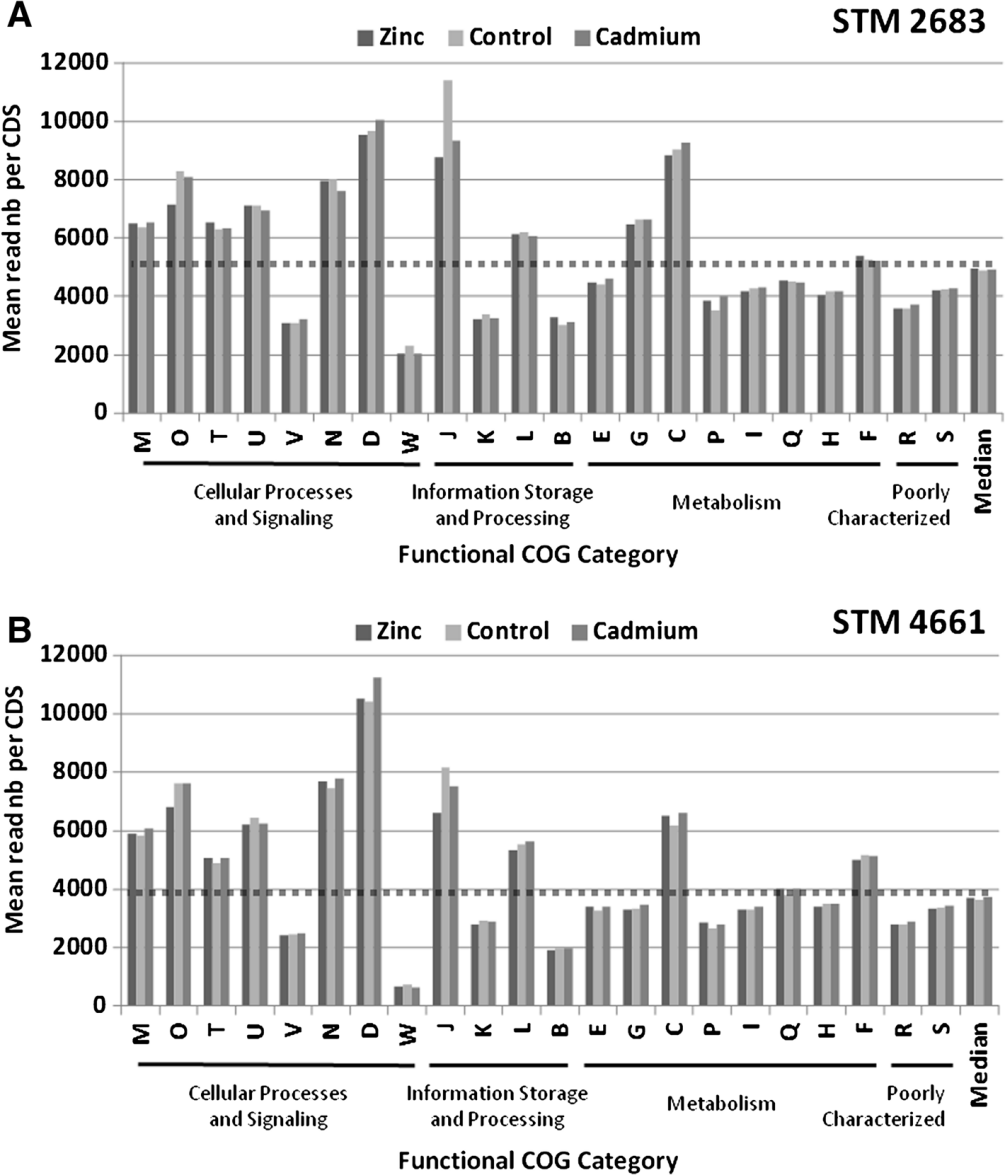
**Transcriptional activity of major functions.** Histograms representing Mean read numbers per COG class for STM 2683 (**A**) and STM 4661 (**B**) in the three treatments (Zinc, Control and Cadmium). The median read numbers per class and per treatment (last bars on the histograms) were used to draw the dotted line above which classes can be considered as transcriptionally over-active and those below as under-active.

^2^ Transcriptionally over-active COG Class for both strains and in all treatments (cf. Figure 4).

**Figure 5:**
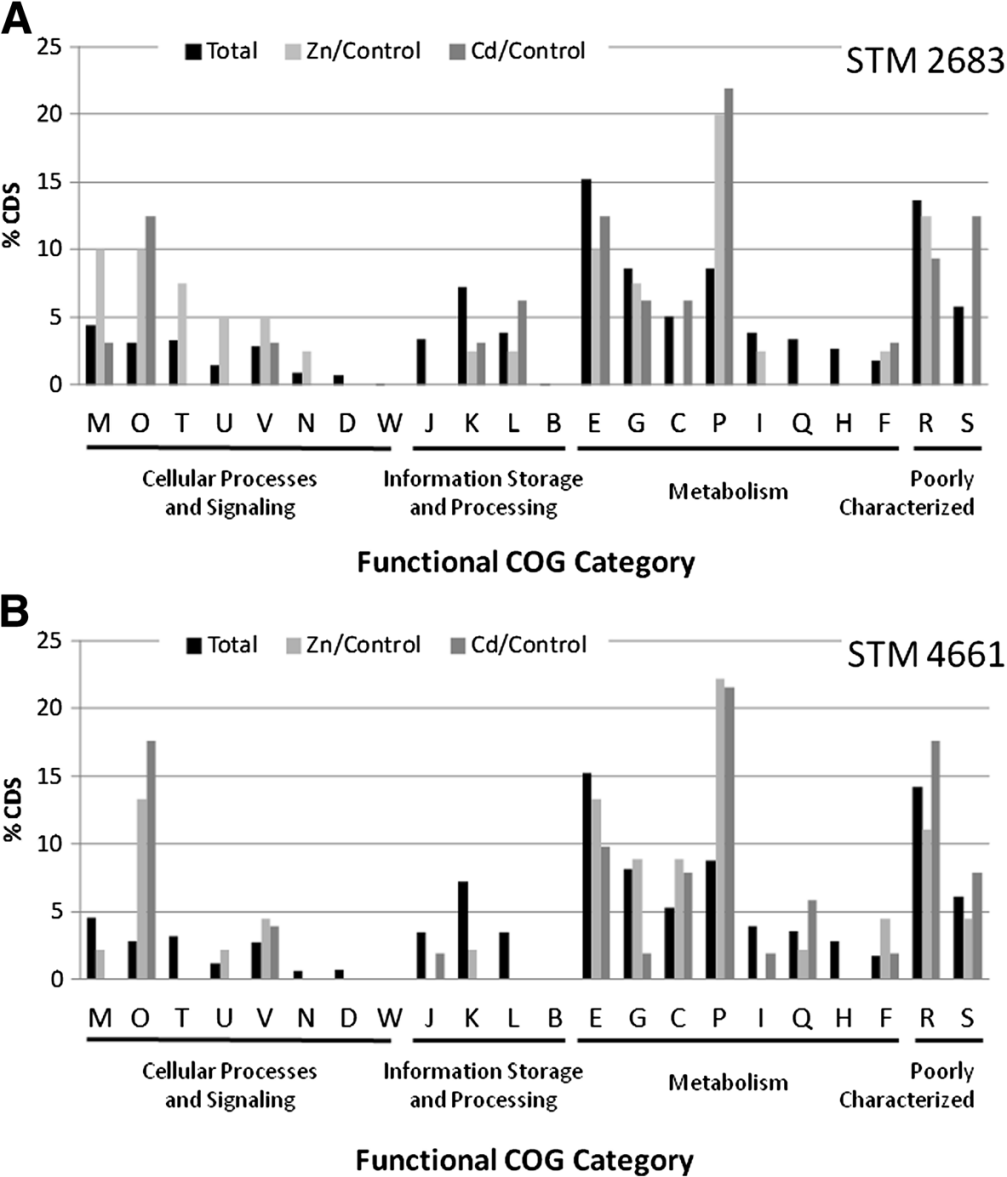
**Impact of metals on the major functions.** Histograms representing the percentages of CDS classified by functional COG category for STM 2683 (**A**) and STM 4661 (**B**) in the total set of CDS (black bars), in the Zinc or Cadmium treatments when compared to the control treatment (respectively light and dark grey bars). For a defined COG class, grey bars above the black ones indicate that upon metal treatment, the corresponding COG class contains a higher proportion of CDS that display differential regulation.

^3^ COG Class presenting a higher proportion of differentially regulated genes for both strains and both metal treatments when compared to control treatments (cf. Figure 5).

To estimate absolute RNA contents in *Mesorhizobium* transcriptomes in relation to functional classes, the sums of reads for the CDS assigned to each COG functional category were calculated for each treatment. The pie charts representing the proportions of each COG class (B-W) in the transcriptomes of *Mesorhizobium* were produced using the means of read numbers for all three treatments (Figure 3A and 3C for STM 2683 and STM 4661, respectively). For all the 6 transcriptomes obtained by RNAseq (2 isolates and 3 treatments), the median read number per COG class was calculated to distinguish the most represented classes at the quantitative level in the transcriptomes (Figure 3B and 3D for STM 2683 and STM 4661, respectively). The most represented functional categories at the transcriptional level in both isolates belonged to metabolism processes, principally COG classes E, G, P and C which refer to the metabolism and transport of amino acids, carbohydrates, inorganic ions and energy production and conversion, respectively, the poorly characterized COG class R, and class J which refers to translation and ribosome structure and biogenesis. Our quantitative analysis highlights the relative content of each functional class in the transcriptomes likely to be representative of *Mesorhizobium* growth in the medium under study. However, as expected, the number of transcripts per COG class was correlated to the number of CDS per class (R^2^ of 0.8 data not shown) and is thus reflecting the relative genomic content. In order to estimate the relative transcriptional activity of each functional category, the sums of reads per COG class was normalized to their respective CDS numbers (Figure 4). Such normalization shows that the COG classes belonging to (i) cellular processes and signaling (classes M, O, T, U, N and D) (ii) information and storage processing (classes J and L) and (iii) metabolism (class C) could be considered as transcriptionally over-active as compared to the other COG classes in both isolates (Figure 4 and Table 3). These results are not surprising as the cells were harvested during the exponential growth phase which requires the involvement of many genes whose function is associated with (i) basic cellular processes such as cell cycle control, cell division, chromosome partitioning, cell wall/ membrane/ envelope biogenesis, signal transduction mechanisms, intracellular trafficking and (ii) information storage and processing such as replication and translation, all of which require high energy production and conversion.

Interestingly, slight differences were noted for the COG class J that refers to translation, ribosomal structure and biogenesis. Indeed, in both isolates, the two metal treatments triggered a decrease of the transcription levels for genes assigned to this particular COG class (Figure 3 and 4). Box plot representations of the log2 fold changes between metal and control treatment data as classified by COG functional categories (Additional file 9) confirmed this observation, suggesting that metal treatment affects translation and ribosome structure and biogenesis. These results are in line with observations made by Pereira and colleagues (2006) who found that metal exposure could affect protein levels as shown for *Rhizobium leguminosarum* bv. *viciae* isolates [19]. However, despite a relatively high tolerance of our *Mesorhizobium* isolates as compared to other mesorhizobia [7], our transcriptomic data suggest that a global repression of the translation machinery occurs upon Cadmium or Zinc exposure; according to Pereira’s hypothesis, such repression should categorize our isolates as metal sensitive. Additional experiments are required to verify whether our isolates are affected in protein synthesis and compare their levels to other mesorhizobia, including strains more tolerant than those presently studied in order to validate this hypothesis and show that it is applicable to the *Mesorhizobium* genus. Interestingly, previous studies on *E. coli* show that upon Cd exposure, protein biosynthesis machinery was stopped [20].

In order to assess the effect of metals on general functional processes in the isolates, we compared the functional distribution of the most significantly deregulated genes (padj ≤ 0.1) to which a COG class was assigned to full gene set distribution (Figure 5). The percentage of deregulated genes per category gives a rapid mean to estimate which COG classes are the most affected by metal treatment. Interestingly, classes O and P appeared as over-represented when compared to the full gene set for both metals and both isolates. Class O refers to post-translational modification, protein turnover, chaperones, which supports the hypothesis that protein biosynthesis and stability are affected by Zn and Cd exposure. Class P corresponds to inorganic ion transport and metabolism, which indicates that Zn or Cd divalent cations are processed by bacteria as soon as they are sensed, most probably through export systems [9].

### Identification of genes regulated by metals in *Mesorhizobium* strains STM 2683 and STM 4661

Transcriptional profiles obtained using the genome-wide RNAseq approach was performed on pooled samples. The high reproducibility between treatments, as exemplified by the high correlation factors of read counts and relative expression values of the RNAseq datasets allowed us to obtain adjusted *p-values* (FDR) with DESeq. Putative CDS presenting FDR values < 0.1 in a “Metal” *vs* “Control” experiment are listed in Tables 4 and 5 for STM 2683 and STM 4661, respectively. A total of 72/6,844 and 68/6,994 putative CDS (or 1.05% and 0.97% CDS for STM 2683 and STM 4661, respectively) were found to be significantly differentially expressed by one or both metals for STM 2683 and STM 4661, respectively. These putative differentially expressed CDS are classified into six categories: transport, sequestration, regulation, oxidoreduction, others and unclassified. Thirteen out of seventy-two and 13/68 putative CDS (18% for STM 2683 and 19.1% for STM 4661) encoded transport proteins, 15/72 and 16/68 putative CDS (20.8% for STM 2683 and 23.5% for STM 4661) encoded proteins possibly involved in sequestration, 3/72 and 1/68 (4.2% for STM 2683 and 1.5% for STM 4661) encoded regulators, 5/72 and 10/68 putative CDS (7% for STM 2683 and 14.7% for STM 4661) encoded proteins involved in oxidoreduction, 6/72 and 4/68 putative CDS (8.3% for STM 2683 and 5.9% for STM 4661) encoded proteins possibly not involved in the metal response and 30/72 and 24/68 putative CDS (41.7% for STM 2683 and 35.3% for STM 4661) were unclassified for STM 2683 and 4661, respectively. As expected, the category “unclassified” is the most represented among significant regulated putative CDS. Few CDS are classified into the category “others” which can be assimilated to the non-specific metal response, suggesting that the RNAseq approach in our conditions is reliable and allowed us to identify the genes involved in the specific metal response. “Transport” and “sequestration” are the most represented categories after the “unclassified” category among significantly regulated putative CDS, which is in agreement with the fact that transport and sequestration proteins were found over-represented in the three available metallicolous mesorhizobial genomes. Among the putative CDS significantly regulated in STM 2683, 39 (27 up and 12 down) and 48 (38 up and 10 down) were regulated upon Zn and Cd exposure, respectively; 15 of them were regulated by both metals. In STM 4661, 36 (23 up and 13 down) and 49 (42 up and 7 down) were regulated after Zn and Cd treatments, respectively, and 17 were regulated by both metals (Tables 4 and 5).

**Table 4:** List of putative STM 2683 CDS differentially regulated upon metal exposure

Label*	**CDS description and putative function**	**Zn / Control**		**Cd / Control**		
		**log2 Fold**	**FDR**	**log2 Fold**	**FDR**	**STM 4661 Homologs **^**§**^
***Transport***						
**MESS2v1_p180001**	**Cation efflux system protein czcD-type (fragment)**	**1.97**	**2.5E-16**	**1.41**	**2E-08**	**MESS4v1_360039**
**MESS2v1_300037**	**ABC transporter, periplasmic binding protein, Zn import**	**−4.09**	**2E-60**	**2.54**	**1.3E-24**	**MESS4v1_520016**
**MESS2v1_300038**	**ABC transporter, permease protein, Zn import**	**−4.13**	**3E-116**	**2.49**	**4.5E-43**	**MESS4v1_520017**
**MESS2v1_300039**	**ABC transporter-related protein, Zn import**	**−4.37**	**2E-127**	**2.32**	**4E-24**	**MESS4v1_520018**
**MESS2v1_740030**	**Heavy metal-translocating P-type ATPase CadA**	**3.07**	**6.7E-21**	**2.2**	**2.2E-12**	**MESS4v1_360013**
MESS2v1_980072	Transporter protein of unknown function	3.36	3E-42	2.2	4.9E-17	Not found
**MESS2v1_980073**	**Zinc, cobalt and lead efflux system ZntA**	**4.65**	**2E-134**	**3.48**	**6E-131**	**MESS4v1_610026**
MESS2v1_620035	Multidrug efflux system, subunit C, MdtC	0.93	0.0283	0.13	1	MESS4v1_690033
MESS2v1_620036	Multidrug efflux system, subunit A, MtdA	0.95	0.0116	0.0714	1	MESS4v1_690034
**MESS2v1_730268**	**Putative cation efflux system protein (Cobalt-zinc-cadmium resistance CzcD-like), CDF family (fragment, part 1)**	**1.12**	**0.0182**	**0.67**	**0.69**	**MESS4v1_360039**
**MESS2v1_730269**	**Putative cation efflux system protein (Cobalt-zinc-cadmium resistance CzcD-like), CDF family (fragment, part 2)**	**1.26**	**0.00012**	**0.64**	**0.48**	**MESS4v1_360039**
MESS2v1_320039	ABC transporter, permease protein, sugar transporter	0.44	1	0.82	0.0364	MESS4v1_530098
MESS2v1_730254	Efflux transporter, RND family, MFP subunit	−0.32	1	1.2	9.6E-05	Not found
***Metal sequestration***						
**MESS2v1_p180003**	**conserved and cytoplasmic protein of unknown function, contains 2 CPX Zn finger domain**	**2.01**	**4.4E-17**	**1.42**	**5.5E-08**	**MESS4v1_360040**
**MESS2v1_730267**	**conserved and cytoplasmic protein of unknown function, contains 2 CPX Zn finger domain**	**1.09**	**0.0263**	**0.58**	**1**	**MESS4v1_360040**
MESS2v1_1150003	conserved and cytoplasmic protein of unknown function, contains 2 CPX Zn finger domain	−0.78	0.0792	−0.3	1	Not found
**MESS2v1_310036**	**Cyclic beta-glucan succinyl transferase OpgC, cytoplasmic membrane**	**−0.14**	**1**	**0.76**	**0.0813**	**MESS4v1_520093**
MESS2v1_1210019	Antibiotic biosynthesis monooxygenase, putative, involved in the biosynthesis of extracellular polysaccharides	−0.0992	1	0.77	0.0654	MESS4v1_160035
**MESS2v1_740029**	**Cytochrome c biogenesis protein, transmembrane region CcdA, putative, involved in reduction as Zn or Cd Sulfides**	**3.04**	**6.2E-29**	**2.17**	**1.9E-30**	**MESS4v1_360014**
MESS2v1_740015	Phosphoesterase PA-phosphatase-related (fragment), located in cytoplasmic membrane	1.19	3E-05	0.73	0.23	Not found
**MESS2v1_740019**	**Signal peptidase II. Aspartic peptidase. MEROPS family A08, located in cytoplasmic membrane**	**1.64**	**1.5E-12**	**0.65**	**0.3**	**MESS4v1_360023**
MESS2v1_440031	Rhodanese domain protein, putative, involved in reduction as sulfide	−0.0727	1	0.88	0.0481	MESS4v1_590053
MESS2v1_1160006	Transcription regulator, putative, involved in reduction as Cd Sulfides	0.0363	1	0.93	0.00391	MESS4v1_120164
MESS2v1_1660011	Putative cytochrome c biogenesis protein, involved in reduction as Cd sulfide, located in cytoplasmic membrane	0.1	1	1.59	2.9E-11	Not found
MESS2v1_1660012	Thioredoxin-related, putative, involved in reduction as Cd sulfide	−0.0234	1	1.68	9.2E-12	Not found
MESS2v1_160018	Serine endoprotease, periplasmic DegQ, putative, involved in Zn periplasmic binding	1.7	1.7E-10	0.24	1	MESS4v1_440007
MESS2v1_160022	Glutamate-ammonia-ligase adenylyltransferase GlnE, putative, involved in Zn periplasmic binding and participating to degQ activity	0.94	0.00988	0.33	1	MESS4v1_440011
**MESS2v1_1530017**	**conserved exported protein of unknown function, putative metal-binding protein**	**−0.45**	**1**	**−1.58**	**3.9E-06**	**MESS4v1_830506**
***Regulation***						
MESS2v1_160020	putative Two-component transcriptional regulator; transcriptional regulator involved in heavy-metal (Cu/Zn) homeostasis	1.12	0.00012	0.0744	1	MESS4v1_440009
MESS2v1_160021	Sensor protein	0.87	0.0294	0.17	1	MESS4v1_440010
MESS2v1_300035	Transcriptional regulator, AsnC family	−0.38	1	1.12	0.00047	MESS4v1_520012
***Oxidoreduction***						
MESS2v1_110046	Alpha/beta hydrolase fold protein	−0.87	0.0228	0.18	1	Not found
MESS2v1_620027	NAD(P)H:quinone oxidoreductase	1.1	0.00721	0.19	1	Not found
MESS2v1_1150002	Haloalkane dehalogenase	−0.83	0.0623	−0.74	0.21	Not found
MESS2v1_300036	conserved protein of unknown function, putative, hydrolase activity	−0.77	0.27	1.5	4.2E-07	MESS4v1_520015
**MESS2v1_790137**	**conserved exported protein of unknown function, putative, succinate dehydrogenase/fumarate reductase, flavoprotein subunit**	**−0.26**	**1**	**1.08**	**0.00047**	**MESS4v1_830086**
***Others***						
**MESS2v1_1590041**	**conserved protein of unknown function - N-acetyltransferase activity**	**−0.94**	**0.00626**	**−0.65**	**0.3**	**MESS4v1_830271**
MESS2v1_10049	Protein folding- Stress - chaperonin groES	−0.91	0.0796	−0.51	1	MESS4v1_60077, MESS4v1_430231, MESS4v1_510195
MESS2v1_900005	transposase	−0.0531	1	0.95	0.0027	
MESS2v1_980071	protein of unknown function, putative transposase	0.8	0.0689	−0.0345	1	Not found
MESS2v1_1530018	Nuclear export factor GLE1 (fragment)	−0.4	1	−1.54	2.6E-09	Not found
MESS2v1_1660010	conserved protein of unknown function, putative, antirestriction protein ArdC	−0.0576	1	0.89	0.00928	Not found
***Unclassified***						
MESS2v1_p180002	protein of unknown function	2.02	1.8E-14	1.29	1.1E-06	Not found
MESS2v1_740028	exported protein of unknown function	2	3.1E-28	1.41	5E-07	Not found
MESS2v1_740031	protein of unknown function	2.17	2.4E-39	1.43	1.4E-09	Not found
MESS2v1_1030151	protein of unknown function	−1.5	2.7E-12	−1.75	2.1E-14	Not found
MESS2v1_1280026	protein of unknown function	1.73	6.8E-13	1.82	3.4E-13	Partial MESS4v1_220037
MESS2v1_1520011	protein of unknown function	−0.94	0.0626	−0.99	0.0116	Not found
MESS2v1_160019	protein of unknown function	1.71	1.1E-12	0.27	1	Not found
MESS2v1_280030	protein of unknown function	−0.79	0.0689	−0.66	0.29	Not found
MESS2v1_460011	protein of unknown function	0.79	0.0689	0.13	1	Not found
MESS2v1_620020	conserved exported protein of unknown function	−0.98	0.0834	0.12	1	Not found
MESS2v1_740027	protein of unknown function	1.18	3.5E-05	0.67	0.41	Not found
MESS2v1_980070	protein of unknown function	1.72	8.8E-08	0.92	0.62	Not found
MESS2v1_p110013	protein of unknown function	−0.72	0.73	−1.25	0.0192	Not found
MESS2v1_50011	protein of unknown function	−0.57	0.93	−0.99	0.0167	Not found
MESS2v1_310082	protein of unknown function	0.35	1	0.75	0.0813	Not found
MESS2v1_360012	protein of unknown function	−0.53	1	−0.77	0.0585	Not found
MESS2v1_600022	protein of unknown function	0.28	1	0.89	0.0833	Not found
MESS2v1_600023	conserved exported protein of unknown function	0.33	1	1.1	0.00014	MESS4v1_680074
MESS2v1_660006	protein of unknown function	−0.34	1	−0.75	0.0813	Not found
MESS2v1_730256	conserved protein of unknown function	−0.083	1	0.93	0.0049	Not found
MESS2v1_760133	protein of unknown function	−0.65	0.4	−0.75	0.0833	Not found
MESS2v1_1100024	conserved exported protein of unknown function	−0.41	1	0.94	0.0226	MESS4v1_120015
MESS2v1_1100025	conserved exported protein of unknown function	−0.12	1	1.05	0.00597	MESS4v1_120016
MESS2v1_1100026	conserved exported protein of unknown function	−0.22	1	1.05	0.00426	MESS4v1_120016
MESS2v1_1210020	conserved protein of unknown function	−0.71	0.44	1.36	5.1E-07	MESS4v1_160036
MESS2v1_1210021	conserved protein of unknown function	0.0842	1	1.2	0.00105	MESS4v1_160037
MESS2v1_1270078	conserved exported protein of unknown function	−0.0732	1	0.84	0.0503	MESS4v1_220001
MESS2v1_1530016	protein of unknown function	−0.42	1	−1.52	4.5E-05	Not found
MESS2v1_1660004	membrane protein of unknown function	0.16	1	1.04	0.00032	Not found
MESS2v1_1660005	conserved protein of unknown function	0.38	1	1.5	4.1E-12	Not found

* Gene labels (CDS) were obtained from MaGe; CDS significantly regulated (FDR < 0.1) upon metal exposure in both STM 2683 and STM 4661 are in bold. MESS2v1_XXX are genes located on the chromosome and MESS2v1_**p**XXX are genes located on the plasmid.

^**§**^ When detected, STM 4661 homologs are indicated (See Methods for details).

**Table 5:** List of putative STM 4661 CDS differentially regulated upon metal exposure

Label*	**CDS description and putative function**	**Zn / Control**		**Cd / Control**		
		**log2 Fold**	**FDR**	**log2 Fold**	**FDR**	**STM 2683 Homologs **^**§**^
***Transport***						
**MESS4v1_360013**	**Heavy metal-translocating P-type ATPase CadA**	**5.32**	**1.53E-121**	**3.38**	**1.96E-171**	**MESS2v1_740030**
MESS4v1_360015	conserved membrane protein of unknown function, putative CDF	2.2	1.10E-29	0.88	1.12E-02	Not found
**MESS4v1_520016**	**ABC transporter, periplasmic binding protein, Zn import**	**−2.98**	**4.47E-96**	**4.19**	**9.71E-173**	**MESS2v1_300037**
**MESS4v1_520017**	**ABC transporter, permease protein, Zn import**	**−2.59**	**1.49E-33**	**3.28**	**9.43E-144**	**MESS2v1_300038**
**MESS4v1_520018**	**ABC transporter-related protein, Zn import**	**−2.61**	**1.01E-23**	**3.4**	**2.99E-59**	**MESS2v1_300039**
MESS4v1_670093	Transporter protein of unknown function	−1.75	4.35E-06	−1.44	3.69E-04	MESS2v1_580019
**MESS4v1_360039**	**Zinc efflux system ZitB type**	**1.11**	**2.35E-02**	**0.55**	**0.91**	**MESS2v1_730268 MESS2v1_730269 fissed MESS2v1_p180001 (fragment)**
**MESS4v1_610026**	**Zinc, cobalt and lead efflux system ZntA**	**1.77**	**1.07E-10**	**−0.11**	**1**	**MESS2v1_980073**
MESS4v1_750116	Aliphatic sulphonate ABC transporter	−1.02	1.35E-03	−0.22	1	Not found
MESS4v1_240016	Iron-hydroxamate transporter subunit ; ATP-binding component of ABC superfamily, FhuC	−0.81	0.25	−1.3	7.08E-05	MESS2v1_1320036
MESS4v1_360106	ABC transporter, permease protein	0.13	1	0.87	7.05E-03	Not found
MESS4v1_360107	Transporter protein of unknown function	−0.28	1	0.8	3.68E-02	Not found
MESS4v1_670092	Hemin transport protein HmuS	−0.63	0.66	−0.8	6.41E-02	MESS2v1_580018
***Metal sequestration***						
**MESS4v1_360040**	**Conserved and cytoplasmic protein of unknown function, contains 2 CPX Zn finger domains**	**1.02**	**8.10E-02**	**0.54**	**1**	**MESS2v1_730267, MESS2v1_p180003**
MESS4v1_750294	Xanthine dehydrogenase, Fe-S binding subunit XdhC, located in the cytoplasm	0.86	5.42E-02	0.1	1	Not found
MESS4v1_280090	Methyltransferase type 11, located in the cytoplasm	0.2	1	1.05	1.03E-04	MESS2v1_1480043
**MESS4v1_520093**	**Cyclic beta-glucan succinyl transferase OpgC, cytoplasmic membrane**	**0.15**	**1**	**0.81**	**4.02E-02**	**MESS2v1_310036**
**MESS4v1_360014**	**Cytochrome c biogenesis protein, transmembrane region CcdA, putative, involved in reduction as Zn or Cd Sulfides**	**4.07**	**4.31E-48**	**2.22**	**3.75E-31**	**MESS2v1_740029**
MESS4v1_360049	Thiol:disulfide interchange protein CycY, putative, involved in reduction as Zn or Cd Sulfides, located in the periplasm	1.12	1.07E-04	1.93	1.30E-24	MESS2v1_730262
MESS4v1_360050	Reduction as Zn or Cd Sulfides - putative thioredoxin protein	1.18	8.54E-05	2.14	4.83E-29	MESS2v1_730261
MESS4v1_360052	Putative oxidoreductase protein involved in reduction as Zn or Cd Sulfides	0.91	1.13E-02	1.86	9.13E-25	MESS2v1_730259
MESS4v1_360053	Conserved exported protein of unknown function, putative protein-disulfide isomerase	0.82	4.09E-02	1.71	3.89E-22	MESS2v1_730258
**MESS4v1_360023**	**Signal peptidase II. Aspartic peptidase. MEROPS family A08, located in cytoplasmic membrane**	**1.85**	**1.54E-13**	**0.69**	**0.14**	**MESS2v1_740019**
MESS4v1_160007	Peptide methionine sulfoxide reductase MsrA, putative, involved in reduction as Cd Sulfides	8.49E-02	1	1.13	3.01E-04	MESS2v1_1200004
MESS4v1_360103	Glutathione S-transferas, putative, involved in reduction as Cd Sulfides	−0.26	1	0.98	1.81E-03	Not found
MESS4v1_310058	Fe(3+)-binding periplasmic protein, FbpA	−0.87	9.42E-02	−1.01	1.37E-03	MESS2v1_1490012
MESS4v1_360047	Conserved exported protein of unknown function, predicted metal-binding domain	1.33	1.93E-07	2.27	1.93E-36	MESS2v1_730264
MESS4v1_360048	Conserved exported protein of unknown function, predicted metal-binding domain	1.24	2.38E-06	2.19	8.73E-21	MESS2v1_730263
**MESS4v1_830506**	**conserved exported protein of unknown function, putative metal-binding protein**	**4.76E-02**	**1**	**−0.9**	**5.15E-02**	**MESS2v1_1530017**
***Regulation***						
MESS4v1_580068	ROK family protein	−0.84	3.37E-02	−0.36	1	MESS2v1_400012
***Oxidoreduction***						
MESS4v1_360054	Cytochrome c-type biogenesis protein CdcA (fragment)	0.92	2.06E-02	1.59	2.89E-18	
MESS4v1_360046	Multicopper oxidase, type 3	1.24	4.42E-06	2.09	1.95E-19	MESS2v1_730265
MESS4v1_750295	Putative Medium FAD-binding subunit of molybdenum enzyme	1.03	1.13E-02	0.29	1	Not found
MESS4v1_310027	Aldo/keto reductase	−3.16E-02	1	0.74	7.75E-02	MESS2v1_1480132
MESS4v1_340203	Exported protein of unknown function, putative hydrolase	−3.91E-02	1	0.84	4.40E-02	Not found
MESS4v1_340204	Conserved protein of unknown function, putative hydrolase	−5.32E-02	1	0.78	6.89E-02	Not found
MESS4v1_360100	Putative dioxygenase	−0.25	1	0.98	4.02E-03	Not found
MESS4v1_360104	3-hydroxybutyryl-coA dehydrogenase	−0.22	1	0.8	4.28E-02	Not found
MESS4v1_820066	Putative reductase	−0.1	1	0.88	1.07E-02	MESS2v1_790060
**MESS4v1_830086**	**conserved exported protein of unknown function, putative succinate dehydrogenase/fumarate reductase, flavoprotein subunit**	**−0.21**	**1**	**2.18**	**2.63E-32**	**MESS2v1_790137**
***Others***						
**MESS4v1_830271**	**conserved protein of unknown function - N-acetyltransferase activity**	**−1.08**	**3.12E-04**	**−0.47**	**1**	**MESS2v1_1590041**
MESS4v1_830272	putative ornithine decarboxylase	−0.98	1.42E-02	−0.62	0.32	MESS2v1_1590040
MESS4v1_580067	Tagatose-1,6-bisphosphate aldolase, located in the cytoplasm	−0.94	1.87E-02	−0.4	1	MESS2v1_400011
MESS4v1_360102	Putative translation initiation inhibitor, yjgF family	−0.22	1	0.98	1.84E-03	Not found
***Unclassified***						
MESS4v1_360051	protein of unknown function	0.94	1.42E-02	1.8	3.06E-15	Not found
MESS4v1_280043	conserved protein of unknown function	0.89	3.06E-02	0.38	1	Not found
MESS4v1_330155	conserved protein of unknown function	0.83	8.89E-02	0.59	0.51	Not found
MESS4v1_430174	conserved protein of unknown function	0.88	2.49E-02	0.17	1	MESS2v1_130027
MESS4v1_610025	protein of unknown function	1.04	2.09E-03	−4.88E-02	1	Not found
MESS4v1_690015	protein of unknown function	−1.64	1.71E-06	0.18	1	Not found
MESS4v1_720075	protein of unknown function	0.84	9.38E-02	0.43	1	Not found
MESS4v1_830036	conserved exported protein of unknown function	−0.89	9.38E-02	0.32	1	MESS2v1_790093
MESS4v1_110143	conserved exported protein of unknown function	2.75E-02	1	0.76	4.41E-02	MESS2v1_1080055
MESS4v1_210104	protein of unknown function	0.46	1	0.76	6.06E-02	Not found
MESS4v1_230034	protein of unknown function	−0.19	1	0.83	2.10E-02	Not found
MESS4v1_240036	conserved exported protein of unknown function	1.38E-03	1	0.75	6.82E-02	Not found
MESS4v1_360025	protein of unknown function	0.2	1	0.83	4.41E-02	Not found
MESS4v1_360101	conserved protein of unknown function	−0.17	1	0.92	6.17E-03	Not found
MESS4v1_360109	protein of unknown function	−0.12	1	1.03	1.37E-03	Not found
MESS4v1_490002	conserved protein of unknown function	0.12	1	0.78	6.89E-02	MESS2v1_210010
MESS4v1_560009	protein of unknown function	−7.77E-02	1	0.77	4.07E-02	Not found
MESS4v1_560010	conserved exported protein of unknown function	−0.34	1	0.88	6.33E-03	Not found
MESS4v1_640142	protein of unknown function	−0.57	0.69	−0.82	3.68E-02	Not found
MESS4v1_680031	protein of unknown function	−0.69	0.25	−0.78	5.49E-02	MESS2v1_590015
MESS4v1_710103	conserved membrane protein of unknown function	−0.33	1	0.75	7.73E-02	MESS2v1_650084
MESS4v1_750195	conserved protein of unknown function	0.32	1	0.81	2.31E-02	MESS2v1_730184
MESS4v1_830287	conserved protein of unknown function	−8.46E-02	1	0.76	6.14E-02	Not found
MESS4v1_110128	conserved membrane protein of unknown function	−0.82	3.80E-02	0.56	0.63	MESS2v1_1080036

* Gene labels (CDS) were obtained from MaGe; CDS significantly regulated (FDR < 0.1) upon metal exposure in both STM 2683 and STM 4661 are in bold. MESS4v1_XXX are genes located on the chromosome and MESS4v1_**p**XXX are genes located on the plasmid.

^**§**^ When detected, STM 2683 homologs are indicated (See Methods for details).

Among the 1.05% and 0.97% putative CDS that are significantly and differentially expressed for STM 2683 and STM 4661, respectively, 13 putative CDS presenting homologs for both isolates were identified as significantly regulated under Zn and/or Cd treatments. Among these conserved and significantly regulated putative CDS, some had homologies to genes encoding proteins whose function could be associated to specific metal responses like metal sequestration or metal transport.

### Conserved and differentially regulated genes possibly involved in sequestration

Metal sequestration can be used by microorganisms to reduce metal bioavailability and thereby avoid metal toxicity. Metal sequestration includes cell wall components, periplasmic or cytoplasmic binding proteins involved in the precipitation of metals by phosphates or in their reduction as sulfides, and intracellular binding proteins containing CPX-Zn finger domains.

Among the 13 putative CDS present and regulated by metal(s) in both isolates, several could be involved in extracytoplasmic sequestration. Indeed, we identified a putative *opg*C gene encoding a membrane protein required for succinylation of periplasmic glucans in *M. loti*[21]. It was slightly but significantly induced upon Cd exposure (> 1.6 fold) in both *Mesorhizobium* strains (MESS2v1_310036 in STM 2683 or MESS4v1_520093 in STM 4661) but was not significantly regulated by Zn (Tables 4 and 5). The addition of succinyl groups confers global negative charges to periplasmic cyclic glucans and may thus limit the diffusion of toxic cations into the cytoplasm through immobilization in the bacterial cell membrane. Amongst the conserved and regulated CDS that may be involved in metal precipitation, we also identified a putative signal peptidase II, also referred to as aspartic peptidase, and MEROPS family A08 (MESS2v1_740019 in STM 2683 and MESS4v1_360023 in STM 4661) significantly up-regulated upon Zn exposure (> 3 fold) in both isolates. It presents similarities to the polyprotein peptidase PbrC of *C. metallidurans* strain CH34 that could participate in the precipitation of Pb^2+^ via the generated phosphate ions thus limiting its re-entry into the cell cytoplasm after extrusion via the P_IB_-type ATPase PbrA [22]. Interestingly, a putative membrane-associated phospholipid phosphatase (MESS2v1_740015 in STM 2683) located in the vicinity of the putative signal peptidase II, aspartic peptidase, MEROPS family A08 was also significantly up-regulated by Zn in STM 2683 (> 2 fold). Precipitation of metals such as Cd by surface polysaccharides, phosphates, sulfides or others to form insoluble salts that reduce metal bioavailability has been observed in several microorganisms [23-25]. Additionally, RNAseq data allowed us to identify a few proteins with a thioredoxin domain significantly up-regulated by Cd in STM 2683 (MESS2v1_1660012) and up-regulated by both metals in STM 4661 (MESS4v1_360050). Thioredoxin is a general protein disulfide reductase believed to serve as a cellular antioxidant by reducing protein disulfide bonds produced by various oxidants and also interact with other proteins to form functional protein complexes [26]. A recent study has demonstrated the role of thioredoxin in the reduction of U(VI) to U(IV) and Cr(VI) to Cr(IV) in *Desulfovibrio desulfuricans* G20 [27]. Among our RNAseq data, we identified other CDS significantly up-regulated in the two *Mesorhizobium* strains under Zn and/or Cd exposure (7 CDS identified in STM 2683 and 8 in STM 4661) that may be involved in the precipitation of metals by phosphates or their reduction as sulfides (Tables 4 and 5).

Among the putative CDS possibly involved in metal sequestration, we also identified several conserved hypothetical proteins that contained two cytoplasmic CPX-Zn finger domains (MESS2v1_730267 in STM 2683 and MESS4v1_360040 in STM 4661) that were significantly up-regulated (> 2 fold) upon Zn exposure in both *Mesorhizobium* strains. CPX-Zn finger domains are known to coordinate Zn ions with a combination of cysteine and histidine residues. Interestingly, another homolog was also found in STM 2683 (MESS2v1_p180003), which was significantly induced by both metals (> 4 fold by Zn and > 3 fold by Cd). Several studies suggest a role of cytoplasmic proteins such as Zn-finger proteins or cysteine residues in metal binding. On the cellular speciation of Cd ions in *E. meliloti*, if extracellular sequestration was favored, a significant amount of intracellular metal was however measured [28] which most probably results from chelation. The transcriptomic analyses of *E. coli* in response to a Zn stress showed that adding excess external Zn induced the expression of many genes that are organized in the regulon for cysteine biosynthesis, implying that Zn-binding proteins containing cysteine residues are one of the mechanisms that chelate Zn [29]. Finally, the metallothionein SmtA and several SmtA-like proteins have been described to sequestrate and detoxify Zn and Cd through Zn-fingers in *Synechococcus* PCC7942 , in *P. aeruginosa* and *P. putida*[30].

### Conserved and differentially regulated genes possibly involved in Transport

Metal transport can be used by microorganisms to limit metal entry and thereby limit negative effects on cell components. Metal transport involves efflux proteins such as Resistant Nodulation cell-Division proteins (RND), P_IB_-type ATPases, Cation Diffusion Facilitor proteins (CDF) or Major Facilitor Superfamily systems (MFS) [9], multi-drug transporter proteins and proteins involved in metal homeostasis.

#### Identification of candidate genes possibly involved in transport by CDF efflux systems

Among the putative CDS probably involved in metal efflux**,** we identified a putative CDF-type transporter that presented homologies with ZitB, a Zn-specific transporter identified in *E. coli*[31] and CzcD involved notably in Cd and Zn resistance in *C. metallidurans*[32] that was significantly up-regulated (> 2-fold) after Zn treatment in STM 4661 (MESS4v1_360039). CDF transport systems function as cation/proton antiporters and are driven by concentration, chemiosmotic gradient or potassium gradient [33]. Surprisingly, in STM 2683, the homolog was fissed into two separate CDS (MESS2v1_730268 and MESS2v1_730269) that were similarly regulated. Whether these two CDS were still functional in STM 2683 is not known. We also found in STM 2683, a second CDS (MESS2v1_p180001) showing strong homologies to the 5’ end of CzcD that was induced by Zn (4-fold) and Cd (> 2-fold). However, this particular CDS was located in the edge of a contig, and we therefore lack its 3’ end indicating that it could actually be the true homolog of MESS4v1_360039.

#### Identification of candidate genes possibly involved in co-transport of metals and antibiotics

Additionally, we identified two putative CDS *mdt*C and *mdt*A, encoding a permease protein and a membrane fusion protein, respectively (MESS2v1_620035-36 in STM 2683 and MESS4v1_690033-34 in STM 4661). They were significantly induced by Zn in STM 2683 but their homologs in STM 4661 were not significantly up-regulated by metals. Both genes encoded putative RND-type proteins involved in multidrug efflux. In *Lactococcus lactis* and *E. coli*, MdtA confers multiple antibiotic resistances [34]. It was also reported that in *Salmonella*, *mdt*ABC contributes to Cu and Zn resistance, in addition to their role in the resistance to β-lactams, novobiocin and deoxycholate [35]. In *E. coli*, a microarray analysis also demonstrated that the *mdt*ABC operon was up-regulated in response to stress caused by excess Zn, suggesting that metal ions can modulate bacterial resistance to antibiotics [36]. Possible explanations underlying the co-selection process between metals and antibiotics include co-resistance (different resistance determinants present on the same genetic element) and cross-resistance (the same genetic determinant responsible for resistance to antibiotics and metals) [37].

#### Identification of candidate genes possibly involved in transport by P-type ATPase systems

Among the 13 conserved and regulated CDS probably involved in metal efflux, we identified two P_IB_-type ATPases. P_IB_-type ATPase transporters belong to the large superfamily of ATP-driven pumps involved in the transmembrane transport of charged substrates against their concentration gradients [38]. P_IB_-type ATPases are known mechanisms classically used by microorganisms to maintain constant and non-toxic levels of metals in the cytoplasm. Transcriptomic analyses of *E. coli* or *E. meliloti* under Zn and Cd stress showed the high induction of genes encoding P_IB_-type ATPases [36,39]. Exclusion from cells by efflux transporters like the P_IB_-type ATPase ZntA of *Escherichia coli*[40] or CadA of *Staphylococcus aureus*[41] are among the most studied bacterial P_IB_-type ATPases. We identified, in the RNAseq data of the two *Mesorhizobium* strains, a gene encoding a related ZntA (MESS2v1_980073 in STM 2683 and MESS4v1_610026 in STM 4661) which was significantly induced upon Zn exposure (> 25-fold in STM 2683 and > 3-fold in STM 4661). Intriguingly, the predicted ZntA was also strongly induced by Cd (>11-fold) in STM 2683, while its homolog in STM 4661 was not up-regulated, indicating that different regulatory mechanisms could operate in the two strains. In accordance with this assumption, a putative MerR-type regulator with homologies to ZntR was predicted upstream of ZntA in STM 2683, on the opposite strand. Furthermore, we found highly different numbers of putative metal responsive regulators (HmrR) predicted in their genomes. A total of 19 possible HmrR-encoding CDS were predicted in STM 2683, including a ZntR and a MerR homolog which localized close to a locus involved in Mercury transport and reduction. In contrast, only four putative HmrR-encoding genes were predicted in STM 4661, none of them being automatically annotated as ZntR. The large number of predicted HmrR-encoding genes present in STM 2683 suggests a more complex regulation network in this strain when compared with STM 4661 which could explain the different transcription profiles obtained for several metal regulated genes in the two *Mesorhizobium* strains. Another P_IB_-type ATPase named CadA showing homologies to CadA of *S. aureus* (MESS2v1_740030 in STM 2683 and MESS4v1_360013 in STM 4661) was induced by both metals studied (> 8- and 39-fold induction upon Zn exposure and > 4- and 10-fold induction upon Cd exposure in STM 2683 and STM 4661, respectively). CadA has recently been characterized using a functional screening based on heterologous expression of a cosmid library produced from STM 2683 genomic DNA (Maynaud *et al*., unpublished observation). Directed mutagenesis of this metal P_IB_-type ATPase which displays uncommon characteristics confirmed the involvement of the gene in the resistance of *M. metallidurans* STM 2683^T^ to high Zn concentrations. But alone CadA does not confer maximal metal resistance, showing that other mechanisms including those identified in the present study are expected to participate in resistance to Zn and Cd in our metal-resistant mesorhizobial strains. It should be noted that in both STM 2683 and STM 4661 strains, a CDS showing homologies to the transmembrane region of the cytochrome c-type biogenesis protein (MESS2v1_740029 and MESS4v1_360014, respectively), homolog to CcdA of *Bacillus subtilis* or DsbD of *E. coli,* was located downstream of CadA and similarly up-regulated by both metals (> 4-fold). *ccd*A encodes a thiol-disulfide oxidoreductase involved in the cytochrome c-type biogenesis [42]. A *dsb*D-related gene has been identified as a metal-binding motif involved in Copper tolerance in *E. coli*[43]. The precise role such homologs have in our *Mesorhizobium* strains remains to be addressed even though it is tempting to speculate on a possible involvement in metal binding *via* divalent metal cation-sulfide bond formation or in the functioning of the P_IB_-type ATPase by providing a reducing power.

#### Identification of candidate genes possibly involved in transport by ABC transport systems

**Figure 6:**
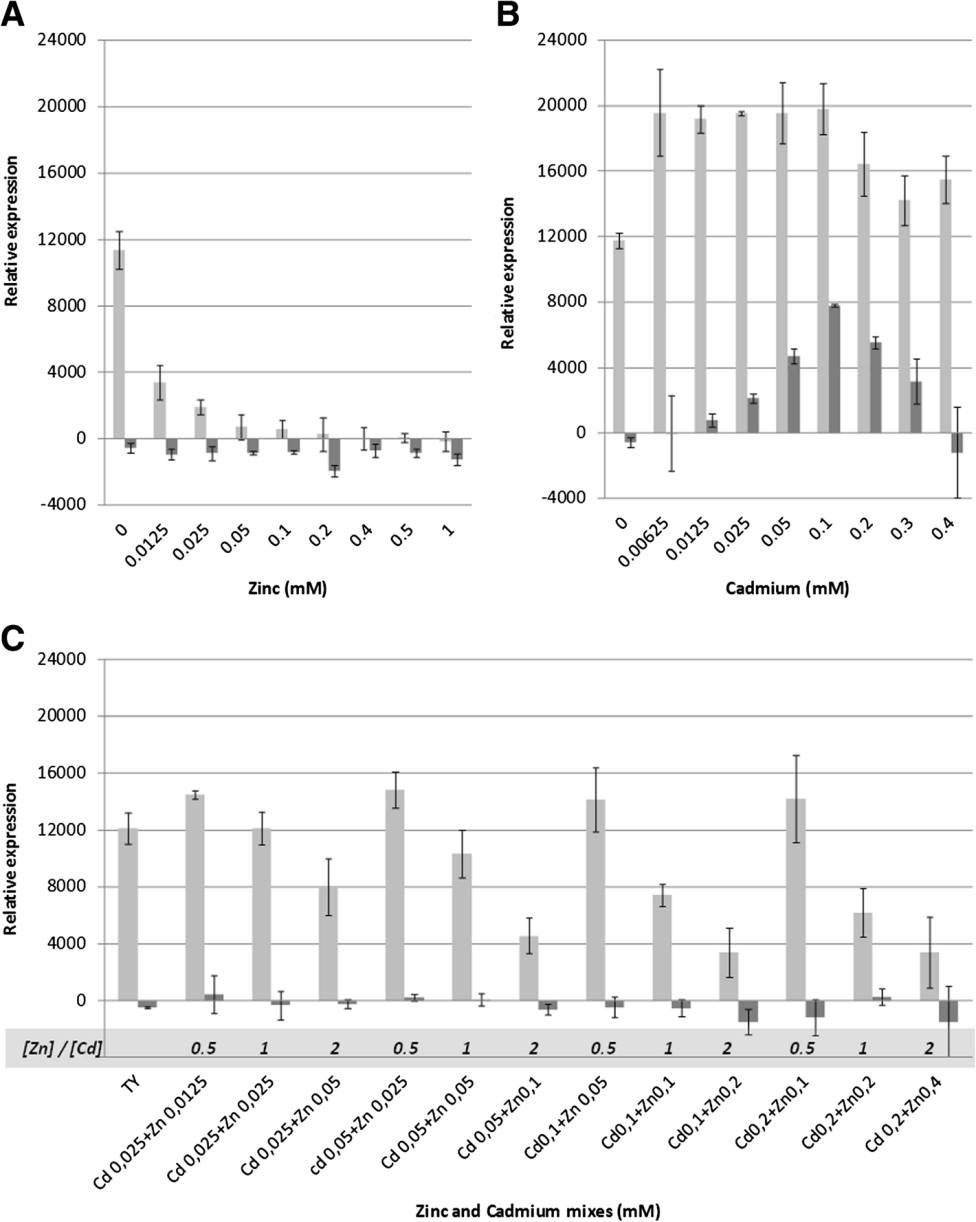
**Effect of zinc and cadmium on the transcriptional activitiy of *znu*C*.*** The effect of increasing concentrations of Zinc (**A**), Cadmium (**B**) or mixes of the two metals (**C**) on the expression of *znu*C using a promoter-PROBE reporter system is shown for STM 2683 (light gray) and the *cad*A mutant (dark gray). [Cd] / [Zn] molar ratios are indicated at the bottom of each bar in **C**. TY medium contained 9.0 μM Zn and no detectable Cd traces.

Among the differentially regulated CDS, we identified three conserved CDS that are organized into an operon (MESS2v1_300039-37 and MESS4v1_520018-16 for STM 2683 and STM 4661 respectively) encoding for an ABC-type transport system. These genes were significantly repressed after Zn exposure (> 17- and > 6-fold repression in STM 2683 and STM 4661, respectively) and induced upon Cd exposure (>5- and >9-fold induction for STM 2683 and STM 4661, respectively). These three CDS contain conserved domains related to ABC-type Mn^2+^/Zn^2+^ transport systems, including respectively the multidomain ATPase component ZnuC (COG1121), the membrane permease ZnuB (COG1108) and the soluble periplasmic Zn-binding protein ZnuA (cd01018) that captures Zn and delivers it to ZnuB. These *znu*CBA orthologs are strongly repressed upon Zn exposure ; therefore they may be involved in Zn homeostasis in our isolates, as showed in *E. coli*[44]. The transcriptional regulation of this high affinity Zn-import system has been shown to be under the control of a Zur (Zn^2+^ Uptake Regulator) repressor that belongs to the Fur (Ferric iron Uptake Regulator) family of transcriptional regulators [44,45]. Interestingly, orthologs of *M. loti* and *E. meliloti* Zur proteins are present in STM 2683 and STM 4661 (MESS2v1_1530029 and MESS4v1_830491, respectively) and *Agrobacterium*-specific conserved *zur* binding sites [46] were also found in the promoter region of the *znu*CBA putative operon in both mesorhizobial strains (data not shown). When bacteria were subjected to Zn in the medium, the *znu*CBA genes were strongly repressed, suggesting, together with high homologies to other bacteria, that the presently identified ATP-binding cassette transport system is also a high affinity Zn-import system involved in Zn homeostasis in *M. metallidurans*. Moreover, previous work showed that other divalent cations such as Fe^2+^, Mn^2+^, Cu^2+^ or Cd^2+^ de-repressed the expression of the *znu* operon in *E. coli*[45]. More recent studies showed that the expression of *znu*A and *znu*B increased upon Cd exposure in *Salmonella* sp. [47] and that ZnuA-tagged proteins increased upon Cd exposure in *E. coli* O157:H7 [48]. This regulation could either be direct (by direct binding of divalent cations to Zur, thereby changing the conformation of the regulator) or indirect (by lowering the intracellular Zn levels), as suggested previously [45,47]. A possible explanation for *znu* induction by Cd could be that direct binding of divalent Cd cations to the Zur regulatory repressor protein as a substitute for Zn alters its conformation and makes it unable to bind DNA, thus directly limiting its repressive activity. Another possibility could be a deficit in intracellular Zn concentration most probably through competition between Zn and other cations for transporters [45,47]. In line with this later observation, we found that both Zn and Cd induced several efflux systems in our isolate (Tables 4 and 5) that may participate in the unspecific export of divalent cations, such as the P_IB_-type ATPase CadA (encoded by MESS2v1_740030 in STM 2683 and MESS4v1_360013 in STM 4661), which is significantly induced by both metals and appears important for Zn adaptation in STM 2683 (Maynaud *et al*., unpublished observations). To increase our knowledge about the regulation of this high-affinity Zn-import system, we used a promoter-PROBE reporter system [49] and analyzed the transcriptional activity of the *znuC* (MESS2v1_300037) in the presence of various metal concentrations and mixes in the wild type STM 2683 and in the *cad*A deleted mutant. We showed that the mutant was affected in Zn tolerance as previously described (Maynaud *et al*., unpublished observation). We tested whether a reduction of the efflux components in STM 2683 through *cad*A mutation could alter the regulation of *znuC* upon exposure to Zn/Cd or to various mixes of the two metals (Figure 6). Increasing the Zn concentration in the medium reduced significantly the relative expression of *znuC* in the wild-type bacterium, reaching non detectable levels when 0.4 mM Zn was used, while no transcriptional activity was detected in the *cad*A mutant (Figure 6A). Since no Cd was present in the control TY medium, the fact that no induction was observed in the *cad*A mutant, while being relatively highly induced in the wild type, indicates that intracellular Zn concentrations were high enough in the mutant to repress *znu*C transcription, and this may result from its lower efflux capacity. When Cd was added to the growth medium, we found that *znu*C expression increased in the wild-type bacterium in the presence of as low as 0.00625 mM, but higher Cd concentrations did not further increase its expression levels and even tended to decrease them when Cd concentration were above 0.2 mM (Figure 6B). Surprisingly, *znu*C expression required higher Cd concentrations in the mutant, reaching a maximum at 0.1 mM Cd and then decreasing again. These results support a more indirect effect of Cd ions which could reduce intracellular Zn levels, leading to a higher stimulation of the high-affinity Zn-import system. To test whether Cd competed directly with the Zur regulator, the effect of various Cd/Zn mixes on *znu*C transcriptional activity was studied (Figure 6C). No transcriptional activity was observed in the *cad*A deletion mutant suggesting that intracellular Zn concentrations were high enough in that strain to repress *znu*C transcription and out-competed the Cd level which, furthermore, was probably extruded or sequestered *via* other mechanisms. Yet, interestingly we found that the molar ratio of Zn and Cd ions in the medium affected *znu*C transcription in the wild-type bacterium. Indeed, despite a metal-dose dependent transcriptional activity of the efflux pump CadA (Maynaud *et al*., unpublished observation), increasing Zn/Cd molar ratios from 0.5 to 2 negatively affected the transcriptional activity of *znu*C in STM 2683, regardless of the total metal concentration used. These results suggest that the two divalent cations compete for binding to the Zur regulator which consequently alters the regulation of *znu*C. Our *znu*C transcriptional data indicate that Cd generates both direct and indirect effects on *znu*CBA regulation in STM 2683, but additional experiments are required to decipher this complex regulation and the effects of Cd on Zn homeostasis.

## Conclusions

In the present study, we used comparative genomic and transcriptomic analyses to study the gene expression profiles associated to Zn and Cd exposure and to identify putative genes involved in metal tolerance in two metallicolous *Mesorhizobium* strains isolated from *Anthyllis* nodules from two distinct mining sites in France. A RNAseq-based approach allowed for the rapid discovery of homologous genes that responded specifically to metals in both strains. Analyses of the genes identified as significantly regulated suggest that transport and sequestration are the most important mechanisms underlying the metal response in metallicolous mesorhizobial strains. We identified proteins involved in metal homeostasis (ABC-type transport system ZnuABC), metal or multi-drug efflux systems which allow for intracellular detoxification (CadA, related ZntA, related CzcD/ZitB, related MdtC and MdtA) and several cell membrane components, periplasmic and cytoplasmic proteins possibly involved in metal precipitation and binding to reduce metal bioavailability. Surprisingly, the number of HmrR regulators and the inductibility of ZntA differed between our two *Mesorhizobium* strains. Additionally, the comparative RNAseq-based approach revealed a low number of genes significantly regulated in both strains (around 1%) and a low number of genes involved in the non-specific metal response, indicating that the approach was fit for identifying genes that specifically respond to metals. Global RNAseq analyses suggested that Zn and Cd also repressed the translational machinery. Our study allowed to detect genes that are conserved among two metallicolous mesorhizobia isolated from distant sites, whose expression is metal-dependent and which show a potential involvement in metal tolerance. Studies on the distribution of potential genetic determinants identified in the present work in other metal tolerant and sensitive *Anthyllis* mesorhizobia will enable the definition of biomarkers. Such candidates may help us to select appropriate rhizobial symbionts to be used as biofertilizers to improve phytostabilization strategies and limit the impacts of mine spoil dispersal on human health and surrounding environments. Finally, functional studies on the molecular determinants identified herein and their speciation during in situ symbiotic growth will allow to evaluate their implication in the metal tolerance of our isolates and their role in symbiotic plant growth promotion on contaminated sites.

## Methods

### Microbiological methods

The bacterial strains and plasmids used in the present study are listed in Additional file 10. The zinc- and cadmium-resistant *Mesorhizobium* species *M. metallidurans* STM 2683^T^ and *Mesorhizobium* sp. STM 4661 were isolated from nodules of a metallicolous ecotype of *Anthyllis vulneraria* originating from Zn-Pb Avinières mine (St Laurent-le-Minier, Cévennes, France) [7] and Eylie mine (Pyrénées Ariégeoises, France), respectively. The complete 16S rRNA sequence of STM 4661 contains one different nucleotide compared to those of STM 2683, suggesting that STM 4661 belongs to a species very closed to *M. metallidurans* and may belong to a new *Mesorhizobium* species as house-keeping genes used for taxonomy do not fit with already described *Mesorhizobium* species (data not shown). The metal-sensitive species *Mesorhizobium* sp. STM 2682 was isolated from the unpolluted soil of the Prunarède site [5]. *Mesorhizobium tianshanense* type strain ORS 2640^T^ was used as a metal-sensitive control. *Mesorhizobium* strains were routinely cultured at 28°C in Tryptone Yeast (TY) medium [50]. Liquid TY contained 9.0 μM of Zn and no Cd and Pb traces after measurement by atomic absorption spectrometry (detection limits <0.06 μM Cd and <0.005 μM Pb). *E. coli* strains were grown at 37°C in Luria-Bertani medium [51]. Gentamycin (Gm, 10 μg ml^-1^), tetracycline (Tc, 15 μg ml^-1^), kanamycin (Km, 50 μg ml^-1^), ampicilline (Ap, 50 μg ml^-1^), and 5-bromo-4-chloro-3-indolyl-beta-D-galactosidase (X-gal, 80 μg ml^-1^) were added to the media as required. Solid media contained 2% agar.

Zn and Cd Minimal Inhibitory Concentrations (MIC) were determined in triplicate on TY liquid medium containing increasing concentrations of ZnSO_4_ (0.5, 1, 1.5, 3, 6, 9, 12, 15, 18, 21 and 24 mM) or CdCl_2_ (0.025, 0.05, 0.1, 0.2, 0.3, 0.4, 0.5, 1, 1.5, 2 and 3 mM) in 96-well microtiter plates. Growth was assessed by Optical Density (OD) at 600 nm until one week incubation at 28°C with shaking (450 rpm) using a spectrofluorometer plate reader (TECAN Infinite M200).

### STM 2683 and STM 4661 genome sequencing, assembly and automated annotation

Genomic DNA from strains STM 2683 and STM 4661 were prepared from 15-ml cultures following previously described protocols [52]. The complete genome sequences of strains STM 2683 and STM 4661 were determined using 454 Titanium technology. A mate-pair genomic library of 8 kb was constructed using Roche reagents from genomic DNA fragmented with HydroShear system. Then sequencing was carried out on this library, giving reads of 350bp average length, up to a final coverage of 30× for STM 4661 and 33× for STM 2683. Assembling was done using Newbler (2.3 version), and a comparison with *Mesorhizobium loti* genome was performed to identify the scaffold organization. The draft genome sequences were uploaded into the MicroScope platform [13] and subjected to the automatic annotation pipeline. The genome sequences are available and can be browsed at the MaGe interface [53] and are part of the “Rhizoscope” project [54].

### STM 2683 and STM 4661 Transcriptomes, RNA purification, sequencing and RNAseq analyses

For the RNAseq analysis of *Mesorhizobium* strains STM 2683 and STM 4661, six biological replicates were pre-grown in TY medium up to mid-exponential phase (up to OD_600nm_ = 0.5) in 250-ml Erlenmeyer flasks at 28°C and 145 rpm. When the pre-cultures reached the mid-exponential phase (after *ca* 16 hours), 15 ml were added to an equal volume of pre-warmed TY containing either nothing (Control treatments), Zn at 1 mM (Zn treatments, 0.5 mM final concentration) or Cd at 0.05 mM (Cd treatments, 0.025 mM final concentration) and were further incubated for 5 hours, which corresponds to *ca* half a generation time, at 28°C at 145 rpm. After 5 hours incubation, 1/10^th^ volume of ice-cold stop buffer (5% phenol in ethanol) was added to each culture and directly centrifuged at 4°C, 8,000 rpm for 4 minutes. Supernatants were quickly discarded and the tubes were re-centrifuged for 1 minute so as to collect all the liquid at the bottom of the tubes which was discarded by pipetting. Cells were snap-frozen in liquid nitrogen and stored in the −80°C freezer until use.

Total RNAs were purified from each replicate, each treatment and each strain individually using the RiboPure™ kit (Ambion) following the manufacturer’s recommendations. After isolation of total RNA, a DNase I treatment was performed as described in the RiboPure™ Bacterial kit protocol. Equal amounts of total RNAs from the biological replicates belonging to the same strain and the same treatment were pooled and subjected to two successive runs of ribosomal RNA subtractions using the Microbe Express™ kit (Ambion) following the manufacturer’s instructions. The rRNA removal procedure consists in incubating RNA samples with magnetic beads to which complementary 16S and 23S rRNA conserved sequences are linked. After hybridization, captured 16S and 23S rRNAs are pulled to the side of tube with magnets allowing unbound mRNAs to be eluted. After two rounds of subtraction, mRNA samples were finally precipitated with ethanol and resuspended in nuclease free water. Total and messenger RNA quantities and quality were assessed by Nanodrop and Bioanalyser using RNA chips for Agilent 2100 Bioanalyser (Additional file 1).

For RNA sequencing, six cDNA libraries corresponding to the three treatments (control, Zn and Cd), 100 ng of mRNA were used according to the Illumina kit instructions. Briefly, the first step in the workflow involves fragmentation of mRNA into small pieces using divalent cations under high temperature. Then the cleaved RNA fragments were copied into first-strand cDNA using reverse transcriptase and random primers. This was followed by second-strand cDNA synthesis using DNA Polymerase I and RNase H. These cDNA fragments then went through an end-repair process, the addition of a single ‘A’ base, and then ligation of the adapters. These products were then purified on a gel to select a size range at 200 pb and enriched by PCR to create the final cDNA library. The efficacy of the library construction was checked in a quality control step that involved measuring the adapter-cDNA size and concentration on an Agilent DNA 1000 chip. Sequencing libraries were denatured with sodium hydroxide and diluted to 6 pM in hybridization buffer for loading onto a single lane of an Illumina HiSeq 2000 flowcell V1.5. Cluster formation, primer hybridization and single-read, 36 sequencing cycles were performed on cBot and HiSeq2000 (Illumina, San Diego, CA) respectively.

After sequencing, the sequences or reads that passed the quality filter were mapped on the annotated genomic objects identified in the genomes of STM 2683 and STM 4661 using ssaha2 software [55]. Mapped reads were converted to read counts per gene using BEDtools [56], which were then analyzed using DESeq standard protocol for conditions with no replicate [16]. DESeq generated differential expression values (fold changes) for all genomic objects and all pairwise comparisons of conditions and associated adjusted *p-values* (padj) controlling for the false discovery rate (FDR) to them. Raw and analyzed RNAseq data are accessible at the MicroScope web interface [15] (Additional file 2).

The distributions of (i) the raw read count numbers per CDS after log10 transformation for each treatment (Additional files 3 and 5) and (ii) the fold changes after normalization and log2 transformations for each intra-species comparison produced by DESeq (Additional files 4 and 6) were analyzed using Microsoft Excel and XLSTAT^TM^ visualization tools such as box plots, histograms, scatter plots or pie charts. The functional COG classification of all CDS in the two genomes was done automatically using COGNiTOR which is included in the automated annotation MicroScope pipeline. CDS with assigned COG classes were retrieved from MicroScope and used to estimate the general composition of the transcriptomes of the two strains as well as the over-transcribed classes. The global effects on the major functional categories Zn and Cd treatments had on the two strains were assessed by comparing the distributions of CDS fold changes classified by COG categories and by comparing COG classes to which the most significantly regulated genes (FDR < 0.1 in at least one comparison) were associated (Additional file 9).

### Real time PCR on selected genes

The primers used for quantitative PCR experiments are listed in Additional file 11. For each technical replicate, 1μg of total RNA from either total pooled RNA used for RNAseq or total RNA isolated from independent biological samples prior to pooling were reverse-transcribed with 400 U of Super-Script II (Invitrogen) and random hexamer primers. qPCRs were performed on a Strategene MXP3005P system using Power SYBER green master mix (Applied Biosystems). PCR started with 10 min incubation at 95°C, followed by 40 cycles consisting of 15 seconds at 95°C and 30 seconds at 60°C. Primer specificity and the formation of primer dimers were monitored by dissociation curves. The expression levels of the metal-translocating P_IB_-type ATPase encoding genes (*cad*A) (MESS2v1_740030 and MESS4v1_360013), the periplasmic binding protein of the ABC-type transporter genes (*znu*A) (MESS2v1_300037 and MESS4v1_520016) and the putative Signal peptidase II genes (MESS2v1_740019 and MESS4v1_360023) were standardized by using *recA* (encoding part of the DNA recombination and repair system) (MESS2v1_330003 and MESS4v1_540005) and *glnA* (encoding part of the glutamine synthetase I) (MESS2v1_390006 and MESS4v1_580041) as references. PCR efficiency (E) for each amplicon was calculated using the linear regression method on the log (fluorescence)-*per*-cycle-number data using Stratagen MXpro software. All qPCRs were performed in three technical replicates using either total pooled RNA used for RNAseq (two replicates for each mesorhizobial strain) or total RNA isolated from three biological samples prior to pooling so as to estimate biological variability (three replicates on STM 2683). Data for each sample were expressed relative to the expression levels of *rec*A or *gln*A by using the mathematical model described previously, which determines the relative quantification of a target gene in comparison to a reference (ref) gene between treatment and control samples [57]. The relative expression ratio (R) of a target gene is calculated based on efficiency (E) and the threshold cycle (Ct) of an unknown sample versus a control and expression in comparison to a reference gene. The average relative quantity for each gene under study was calculated and log2-transformed. Means and standard deviations of these final log2 ratios were calculated using data from three technical replicates. Mean comparison was performed with a multiple comparison of Conover-Iman, using XLSTAT software, to determine significant differences in gene expression levels between inducible genes and reference genes and to determine significant differences between the metal treatments (Cd or Zn condition) and the control metal-free treatments.

### Use of promoter-*gfp* fusion in transcriptional analyses

The promoter region of the ABC transporter ATP-binding protein encoding gene *znu*C (MESS2v1_300039) was amplified using primers STM 2953-Forward and STM 2954-Reverse which generate a 500-bp PCR product. PCR products were cleaned up using illustra™ GFX™ PCR DNA and Gel Band Purification Kits (GE Healthcare). The purified PCR product was ligated into a pGEMt-easy vector (PROMEGA) following the manufacturer’s recommendations to generate plasmid pGEMt-*znu*Cp that were transferred into *E. coli* XL2 Blue Ultra-competent Cells. Inserts from pGEMt-*znu*Cp were excised using the restriction enzyme HindIII and ligated into a pPROBE GT vector which allows for transcriptional fusions with the *gfp* gene [49], previously digested with HindIII and dephosphorylated, and the ligation mix was transferred into electrocompetent *E. coli* strain DH10B. Plasmid DNA was extracted using the Wizard Plus SV Minipreps DNA Purification System kit (Promega) following the manufacturer’s recommendations. Plasmid containing the promoter of *znu*C (pGT-*znu*Cp) was sequenced to verify sequence integrity.

The construction (pGT-*znu*Cp) was transferred into STM 2683 electrocompetent cells as described below. Electro-competent *M*. *metallidurans* STM 2683^T^ cells were prepared following a method developed previously [58] but with slight modifications. Briefly, 5 ml of a fresh bacterial culture were used to inoculate 100 ml of TY medium and incubated at 28°C with shaking at 250 rpm until OD_600nm_ reached 0.5 to 0.7. Cells were harvested by centrifugation at 10,000 rpm for 5 minutes at 4°C. The pellet was washed with 10 ml of ice-cold 300 mM sucrose solution and centrifuged as before. The pellet was finally resuspended in 5 ml of ice-cold 300 mM sucrose containing 15% glycerol. Aliquots of 100–150 μl of electro-competent cells were snap-frozen in liquid nitrogen and stored at −80°C until use. When needed, electro-competent cells were transformed using the following procedure. Cells were thawed on ice and placed in pre-cooled 0.2 mm electroporation cuvettes for 20 minutes on ice. Five to ten μl of 30–50 ng μl^-1^ of plasmid DNA were added to the cuvettes and electroporation was conducted at 12.5 KVolts and 720 ohms. After pulse application, the cell suspensions were diluted with 1 ml of TY medium and incubated at 28°C with shaking for 11hours. After incubation, 100 μl of cells were plated onto TY plates containing appropriate antibiotics. Transformants were selected as single colonies and the presence of the plasmid was verified by PCR.

When needed, STM 2683 carrying the promoter-pPROBE constructs were grown on TY agar plates supplemented with antibiotics. Using sterile toothpicks, fresh colonies were transferred to sterile 8-tube strips containing 100 μl of TY. Cells were homogenized by pipetting and for each transcriptional assay, the same bacterial inocula (10 μl) were used to inoculate 150 μl TY medium supplemented or not with Zn (0.0125, 0.025, 0.05; 0.1, 0.2, 0.4, 0.5 and 1 mM final concentrations) or Cd (0.00625, 0.0125, 0.025, 0.05, 0.1, 0.2, 0.3 and 0.4 mM final concentrations) or mixes of Cd/Zn (0.025/0.0125, 0.025/0.025, 0.025/0.05, 0.05/0.025, 0.05/0.05, 0.05/0.1, 0.1/0.05, 0.1/0.1, 0.1/0.2, 0.2/0.1, 0.2/0.2, 0.2/0.4 mM final concentrations respectively) in 96-well plates. The plates were incubated at 28°C with shaking at 450 rpm. OD_600nm_ and fluorescence (excitation filter at 485 nm and emission filter at 535 nm) were recorded 48 hours post inoculation using a spectrofluorometer plate reader (TECAN Infinite M200). Three transcriptional assays were performed for each bacterial strain carrying the promoter-*gfp* fusion or the promoter-less vector. For the analysis, optical density and fluorescence data were first corrected with the values obtained from the media. Corrected fluorescence values were then normalized to the average OD_600nm_ at each time point.

## Competing interests

The authors declare that they have no competing interests.

## Authors’ contributions

GM prepared RNA samples for RNAseq, performed qPCR, MICs assays, construction of the *gfp*-reporter system and regulation assays and participated in drafting the manuscript. BB, EN and JCCM participated in the analysis and in the writing of the manuscript. DM and MD sequenced and assembled the genomes, ran genomic data through Microscope pipeline and analyzed RNAseq data using DESeq. DS prepared the RNAseq libraires and ran the HiSeq2000 high throughput sequencing. ED performed quality analysis of sequences and the first alignment for controlling the read mappability on the reference genome. ALQ designed the experiments, participated in the preparation of RNA samples for RNAseq, analyzed the data and drafted the manuscript**.** All authors read and approved the final manuscript.

## Supplementary Material

Additional file 1 Qualitative analysis of RNA samples and effect of the rRNA subtractions. Click here for file

Additional file 2 Descriptive statistics of the RNAseq Data. Click here for file

Additional file 3 Histogram representing the log10-transformed read count number per CDS for all treatments. Click here for file

Additional file 4 Histogram representing the log2-fold changes obtained for all CDS and all comparisons. Click here for file

Additional file 5 Scatter plot representations of RNAseq data. Click here for file

Additional file 6 MA plot representations of RNAseq data. Click here for file

Additional file 7 Comparison between quantitative PCR and RNAseq data. Click here for file

Additional file 8 Estimation of the biological variations. Click here for file

Additional file 9 Global effect of the metal treatment on the regulation of genes as classified by COG functional categories. Click here for file

Additional file 10 **Strains, plasmids and primers used **[59]**,**[60]**.** Click here for file

Additional file 11 Primers used for quantitative PCR assays. Click here for file

## References

[B1] Kampa M Castanas E Human health effects of air pollution Environ Pollut 2008 151 2 362 367 10.1016/j.envpol.2007.06.012 17646040

[B2] Nwuche CO Ugoji EO Effects of heavy metal pollution on the soil microbial activity Int J Environ Sci Te 2008 5 3 409 414

[B3] Ghosh M Singh SP A Review on Phytoremediation of Heavy Metals and Utilization of It’s by Products Asian J Energy Environ 2005 6 4 18

[B4] Frérot H Lefebvre C Gruber W Collin C Dos Santos A Escarre J Specific interactions between local metallicolous plants improve the phytostabilization of mine soils Plant Soil 2006 282 1–2 53 65

[B5] Mahieu S Frerot H Vidal C Galiana A Heulin K Maure L Brunel B Lefebvre C Escarre J Cleyet-Marel JC *Anthyllis vulneraria/Mesorhizobium metallidurans*, an efficient symbiotic nitrogen fixing association able to grow in mine tailings highly contaminated by Zn Pb and Cd. Plant Soil 2011 342 1–2 405 417

[B6] Escarre J Lefebvre C Raboyeau S Dossantos A Gruber W Marel JCC Frérot H Noret N Mahieu S Collin C Heavy Metal Concentration Survey in Soils and Plants of the Les Malines Mining District (Southern France): Implications for Soil Restoration Wat Air Soil Pollut 2011 216 1–4 485 504

[B7] Vidal C Chantreuil C Berge O Maure L Escarre J Bena G Brunel B Cleyet-Marel JC *Mesorhizobium metallidurans* sp nov., a metal-resistant symbiont of *Anthyllis vulneraria* growing on metallicolous soil in Languedoc, France Int J Syst Evol Microbiol 2009 59 850 855 10.1099/ijs.0.003327-0 19329619

[B8] Nies DH Microbial heavy-metal resistance Appl Microbiol Biotech 1999 51 6 730 750 10.1007/s002530051457 10422221

[B9] Nies DH Efflux-mediated heavy metal resistance in prokaryotes FEMS Microbiol Rev 2003 27 2–3 313 339 12829273 10.1016/S0168-6445(03)00048-2

[B10] Ma Z Jacobsen FE Giedroc DP Coordination Chemistry of Bacterial Metal Transport and Sensing Chem Rev 2009 109 10 4644 4681 10.1021/cr900077w 19788177 PMC2783614

[B11] Janssen PJ Van Houdt R Moors H Monsieurs P Morin N Michaux A Benotmane MA Leys N Vallaeys T Lapidus A The Complete Genome Sequence of *Cupriavidus metallidurans* Strain CH34, a Master Survivalist in Harsh and Anthropogenic Environments PLoS One 2010 5 5 e10433 10.1371/journal.pone.0010433 20463976 PMC2864759

[B12] Monsieurs P Moors H Van Houdt R Janssen PJ Janssen A Coninx I Mergeay M Leys N Heavy metal resistance in *Cupriavidus metallidurans* CH34 is governed by an intricate transcriptional network BioMetals 2011 24 6 1133 1151 10.1007/s10534-011-9473-y 21706166

[B13] Vallenet D Engelen S Mornico D Cruveiller S Fleury L Lajus A Rouy Z Roche D Salvignol G Scarpelli C MicroScope: a platform for microbial genome annotation and comparative genomics Database (Oxford) 2009 2009 bap021 10.1093/database/bap021 20157493 PMC2790312

[B14] Monchy S Benotmane MA Janssen P Vallaeys T Taghavi S van der Lelie D Mergeay M Plasmids pMOL28 and pMOL30 of *Cupriavidus metallidurans* are specialized in the maximal viable response to heavy metals J Bacteriol 2007 189 20 7417 7425 10.1128/JB.00375-07 17675385 PMC2168447

[B15] MicroScope web interface RNAseq projects http://www.genoscope.cns.fr/agc/microscope/expdata/rnaseqProjects.php

[B16] Anders S Huber W Differential expression analysis for sequence count data Genome Biol 2010 11 10 R106 10.1186/gb-2010-11-10-r106 20979621 PMC3218662

[B17] Benjamini Y Hochberg Y Controlling the False Discovery Rate - a Practical and Powerful Approach to Multiple Testing J Roy Stat Soc B Met 1995 57 1 289 300

[B18] Tatusov RL Natale DA Garkavtsev IV Tatusova TA Shankavaram UT Rao BS Kiryutin B Galperin MY Fedorova ND Koonin EV The COG database: new developments in phylogenetic classification of proteins from complete genomes Nucleic Acids Res 2001 29 1 22 28 10.1093/nar/29.1.22 11125040 PMC29819

[B19] Pereira SI Lima AI Figueira EM Screening possible mechanisms mediating cadmium resistance in Rhizobium leguminosarum bv. viciae isolated from contaminated Portuguese soils Microb Ecol 2006 52 2 176 186 10.1007/s00248-006-9057-5 16897308

[B20] Wang AY Crowley DE Global gene expression responses to cadmium toxicity in *Escherichia coli* J Bacteriol 2005 187 9 3259 3266 10.1128/JB.187.9.3259-3266.2005 15838054 PMC1082819

[B21] Kawaharada Y Kiyota H Eda S Minamisawa K Mitsui H Identification of the *Mesorhizobium loti* gene responsible for glycerophosphorylation of periplasmic cyclic beta-1,2-glucans FEMS Microbiol Lett 2010 302 2 131 137 10.1111/j.1574-6968.2009.01843.x 19951365

[B22] Taghavi S Lesaulnier C Monchy S Wattiez R Mergeay M van der Lelie D Lead(II) resistance in *Cupriavidus metallidurans* CH34: interplay between plasmid and chromosomally-located functions Antonie Van Leeuwenhoek 2009 96 2 171 182 10.1007/s10482-008-9289-0 18953667

[B23] Aiking H Kok K van Heerikhuizen H, van ‘t Riet J Adaptation to Cadmium by *Klebsiella aerogenes* Growing in Continuous Culture Proceeds Mainly via Formation of Cadmium Sulfide Appl Environ Microbiol 1982 44 4 938 944 16346119 10.1128/aem.44.4.938-944.1982 PMC242120

[B24] McEntee JD Woodrow JR Quirk AV Investigation of cadmium resistance in an *Alcaligenes* sp Appl Environ Microbiol 1986 51 3 515 520 3963810 10.1128/aem.51.3.515-520.1986 PMC238911

[B25] Macaskie LE Bonthrone KM Yong P Goddard DT Enzymically mediated bioprecipitation of uranium by a *Citrobacter* sp.: a concerted role for exocellular lipopolysaccharide and associated phosphatase in biomineral formation Microbiology-Uk 2000 146 1855 1867 10.1099/00221287-146-8-1855 10931890

[B26] Zeller T Klug G Thioredoxins in bacteria: functions in oxidative stress response and regulation of thioredoxin genes Naturwissenschaften 2006 93 6 259 266 10.1007/s00114-006-0106-1 16555095

[B27] Li XK Krumholz LR Thioredoxin Is Involved in U(VI) and Cr(VI) Reduction in *Desulfovibrio desulfuricans* G20 J Bacteriol 2009 191 15 4924 4933 10.1128/JB.00197-09 19482922 PMC2715717

[B28] Slaveykova VI Parthasarathy N Dedieu K Toescher D Role of extracellular compounds in Cd-sequestration relative to Cd uptake by bacterium *Sinorhizobium meliloti* Environ Pollut 2010 158 8 2561 2565 10.1016/j.envpol.2010.05.016 20541857

[B29] Yamamoto K Ishihama A Transcriptional response of *Escherichia coli* to external zinc J Bacteriol 2005 187 18 6333 6340 10.1128/JB.187.18.6333-6340.2005 16159766 PMC1236622

[B30] Blindauer CA Harrison MD Robinson AK Parkinson JA Bowness PW Sadler PJ Robinson NJ Multiple bacteria encode metallothioneins and SmtA-like zinc fingers Mol Microbiol 2002 45 5 1421 1432 10.1046/j.1365-2958.2002.03109.x 12207707

[B31] Grass G Fan B Rosen BP Franke S Nies DH Rensing C ZitB (YbgR), a member of the cation diffusion facilitator family, is an additional zinc transporter in *Escherichia coli* J Bacteriol 2001 183 15 4664 4667 10.1128/JB.183.15.4664-4667.2001 11443104 PMC95364

[B32] Anton A Grosse C Reissmann J Pribyl T Nies DH CzcD is a heavy metal ion transporter involved in regulation of heavy metal resistance in *Ralstonia* sp. strain CH34 J Bacteriol 1999 181 22 6876 6881 10559151 10.1128/jb.181.22.6876-6881.1999 PMC94160

[B33] Paulsen IT Saier MH Jr A novel family of ubiquitous heavy metal ion transport proteins J Membr Biol 1997 156 2 99 103 10.1007/s002329900192 9075641

[B34] Perreten V Schwarz FV Teuber M Levy SB Mdt(A), a new efflux protein conferring multiple antibiotic resistance in *Lactococcus lactis* and *Escherichia coli* Antimicrob Agents Chemotherapy 2001 45 4 1109 1114 10.1128/AAC.45.4.1109-1114.2001 PMC90432 11257023

[B35] Nishino K Nikaido E Yamaguchi A Regulation of multidrug efflux systems involved in multidrug and metal resistance of *Salmonella enterica* serovar typhimurium J Bacteriol 2007 189 24 9066 9075 10.1128/JB.01045-07 17933888 PMC2168627

[B36] Lee LJ Barrett JA Poole RK Genome-wide transcriptional response of chemostat-cultured *Escherichia coli* to zinc J Bacteriol 2005 187 3 1124 1134 10.1128/JB.187.3.1124-1134.2005 15659689 PMC545701

[B37] Baker-Austin C Wright MS Stepanauskas R McArthur JV Co-selection of antibiotic and metal resistance Trends Microbiol 2006 14 4 176 182 10.1016/j.tim.2006.02.006 16537105

[B38] Arguello JM Identification of ion-selectivity determinants in heavy-metal transport P1B-type ATPases J Membr Biol 2003 195 2 93 108 10.1007/s00232-003-2048-2 14692449

[B39] Rossbach S Mai DJ Carter EL Sauviac L Capela D Bruand C de Bruijn FJ Response of *Sinorhizobium meliloti* to elevated concentrations of cadmium and zinc Appl Environ Microbiol 2008 74 13 4218 4221 10.1128/AEM.02244-07 18469129 PMC2446505

[B40] Rensing C Mitra B Rosen BP The *zntA* gene of *Escherichia coli* encodes a Zn(II)-translocating P-type ATPase Proc Natl Acad Sci USA 1997 94 26 14326 14331 10.1073/pnas.94.26.14326 9405611 PMC24962

[B41] Nucifora G Chu L Misra TK Silver S Cadmium Resistance from *Staphylococcus*-*Aureus* Plasmid *Pi258* CadA Gene Results from a Cadmium-Efflux ATPase Proc Natl Acad Sci USA 1989 86 10 3544 3548 10.1073/pnas.86.10.3544 2524829 PMC287174

[B42] Fabianek RA Hennecke H Thony-Meyer L Periplasmic protein thiol: disulfide oxidoreductases of *Escherichia coli* FEMS Microbiol Rev 2000 24 3 303 316 10.1111/j.1574-6976.2000.tb00544.x 10841975

[B43] Fong ST Camakaris J Lee BTO Molecular-Genetics of a Chromosomal Locus Involved in Copper Tolerance in *Escherichia coli* K-12 Mol Microbiol 1995 15 6 1127 1137 10.1111/j.1365-2958.1995.tb02286.x 7623666

[B44] Patzer SI Hantke K The ZnuABC high-affinity zinc uptake system and its regulator Zur in *Escherichia coli* Mol Microbiol 1998 28 6 1199 1210 10.1046/j.1365-2958.1998.00883.x 9680209

[B45] Patzer SI Hantke K The zinc-responsive regulator Zur and its control of the znu gene cluster encoding the ZnuABC zinc uptake system in *Escherichia coli* J Biol Chem 2000 275 32 24321 24332 10.1074/jbc.M001775200 10816566

[B46] Panina EM Mironov AA Gelfand MS Comparative genomics of bacterial zinc regulons: Enhanced ion transport, pathogenesis, and rearrangement of ribosomal proteins Proc Natl Acad Sci USA 2003 100 17 9912 9917 10.1073/pnas.1733691100 12904577 PMC187884

[B47] Petrarca P Ammendola S Pasquali P Battistoni A The Zur-Regulated ZinT Protein Is an Auxiliary Component of the High-Affinity ZnuABC Zinc Transporter That Facilitates Metal Recruitment during Severe Zinc Shortage J Bacteriol 2010 192 6 1553 1564 10.1128/JB.01310-09 20097857 PMC2832539

[B48] Gabbianelli R Scotti R Ammendola S Petrarca P Nicolini L Battistoni A Role of ZnuABC and ZinT in *Escherichia coli* O157:H7 zinc acquisition and interaction with epithelial cells BMC Microbiol 2011 11 36 10.1186/1471-2180-11-36 21338480 PMC3053223

[B49] Miller WG Leveau JHJ Lindow SE Improved *gfp* and *inaZ* broad-host-range promoter-probe vectors Mol Plant Microb Interact 2000 13 11 1243 1250 10.1094/MPMI.2000.13.11.1243 11059491

[B50] Beringer JE R factor transfer in *Rhizobium leguminosarum* J Gen Microbiol 1974 84 1 188 198 10.1099/00221287-84-1-188 4612098

[B51] Sambrook J Fritsch EF Maniatis T Molecular Cloning: A Laboratory Manual 1989 2 New York: Cold Spring Harbour Laboratory Press

[B52] Chen WP Kuo TT A simple and rapid method for the preparation of Gram-negative bacterial genomic DNA Nucleic Acids Res 1993 21 9 2260 2260 10.1093/nar/21.9.2260 8502576 PMC309503

[B53] Genome Browser MaGe interfacehttps://www.genoscope.cns.fr/agc/microscope/mage/viewer.php

[B54] MicroScope collaborative projects https://www.genoscope.cns.fr/agc/microscope/about/collabprojects.php?P_id=3

[B55] Ning Z Cox AJ Mullikin JC SSAHA: a fast search method for large DNA databases Genome Res 2001 11 10 1725 1729 10.1101/gr.194201 11591649 PMC311141

[B56] Quinlan AR Hall IM BEDTools: a flexible suite of utilities for comparing genomic features Bioinformatics 2010 26 6 841 842 10.1093/bioinformatics/btq033 20110278 PMC2832824

[B57] Pfaffl MW A new mathematical model for relative quantification in real-time RT-PCR Nucleic Acids Res 2001 29 9 e45 10.1093/nar/29.9.e45 11328886 PMC55695

[B58] Hayashi M Maeda Y Hashimoto Y Murooka Y Efficient transformation of Mesorhizobium huakuii subsp. rengei and Rhizobium species J Biosci Bioeng 2000 89 6 550 553 10.1016/S1389-1723(00)80055-9 16232796

[B59] Durfee T Nelson R Baldwin S Plunkett G III Burland V Mau B Petrosino JF Qin X Muzny DM Ayele M The Complete Genome Sequence of *Escherichia coli* DH10B: Insights into the Biology of a Laboratory Workhorse J Bacteriol 2008 190 7 2597 2606 10.1128/JB.01695-07 18245285 PMC2293198

[B60] Jarvis BDW Van Berkum P Chen WX Nour SM Fernandez MP Cleyet-Marel JC Gilli M Transfer of *Rhizobium loti*, *Rhizobium huakuii*, *Rhizobium ciceri*, *Rhizobium mediterraneum*, and *Rhizobium tianshanense* to *Mesorhizobium* gen. nov Int J Syst Bacteriol 1997 47 3 895 898 10.1099/00207713-47-3-895

