# Supplementary material for: Genome-wide transcriptional responses of two metal-tolerant symbiotic Mesorhizobium isolates to Zinc and Cadmium exposure
Source: BMC Genomics. 2013 Apr 30;14:292. doi: 10.1186/1471-2164-14-292 (PMC3668242; doi:10.1186/1471-2164-14-292)
Supplement: Additional file 1 — Qualitative analysis of RNA samples and effect of the rRNA subtractions. [file 1471-2164-14-292-S1.pptx]

## Slide 1
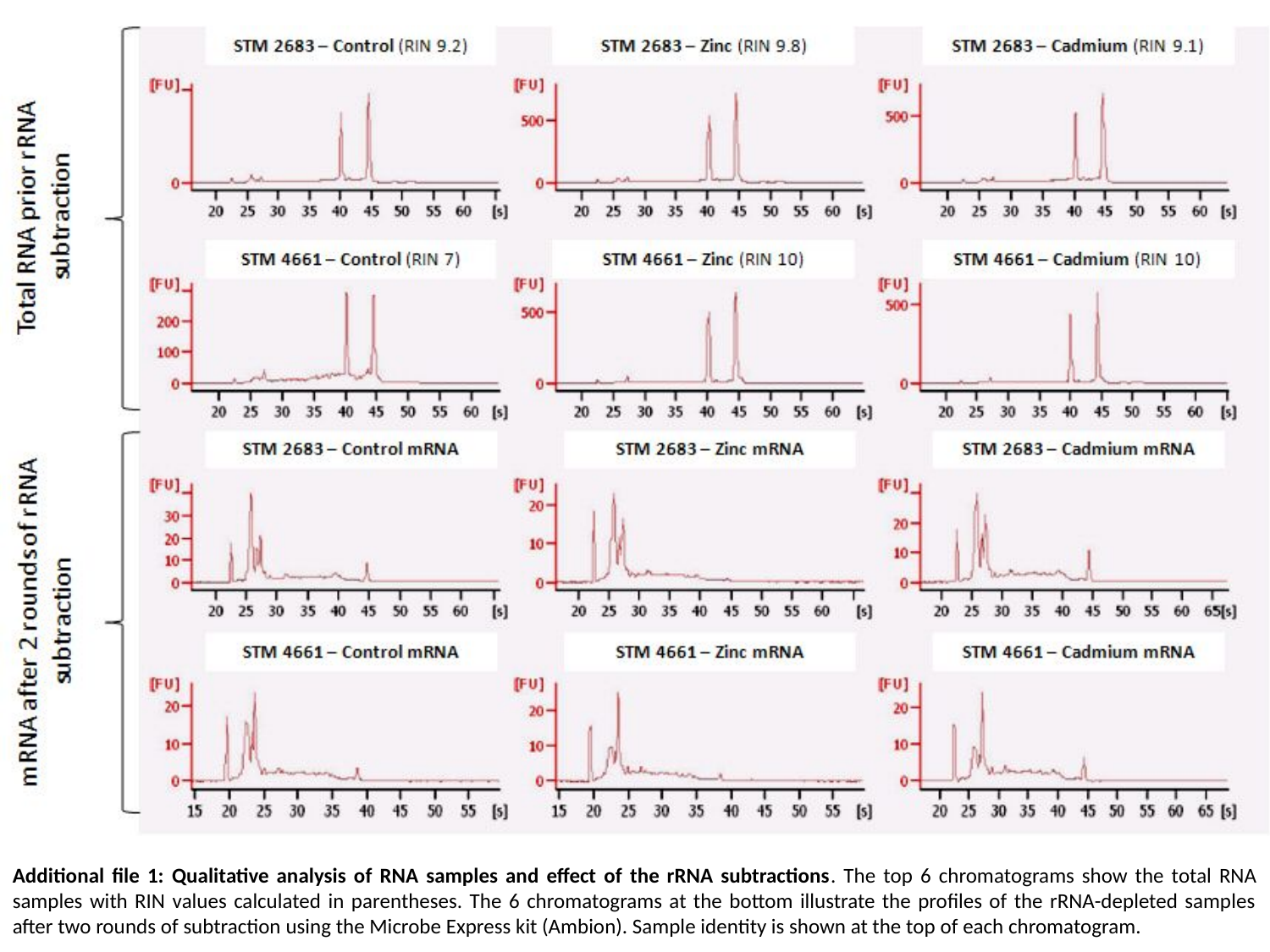

Additional file 1: Qualitative analysis of RNA samples and effect of the rRNA subtractions. The top 6 chromatograms show the total RNA samples with RIN values calculated in parentheses. The 6 chromatograms at the bottom illustrate the profiles of the rRNA-depleted samples after two rounds of subtraction using the Microbe Express kit (Ambion). Sample identity is shown at the top of each chromatogram.
