# Supplementary material for: Genome-wide transcriptional responses of two metal-tolerant symbiotic Mesorhizobium isolates to Zinc and Cadmium exposure
Source: BMC Genomics. 2013 Apr 30;14:292. doi: 10.1186/1471-2164-14-292 (PMC3668242; doi:10.1186/1471-2164-14-292)
Supplement: Additional file 2 — Descriptive statistics of the RNAseq Data. [file 1471-2164-14-292-S2.docx]

**Additional file 2**: Descriptive statistics of the RNAseq Data

| **Read Count Statistics *** | **STM 2683** | | | **STM 4661** | | |
| --- | --- | --- | --- | --- | --- | --- |
|  | **Cadmium** | **Control** | **Zinc** | **Cadmium** | **Control** | **Zinc** |
| **Nb. of observations** | 6908 | 6908 | 6908 | 7065 | 7065 | 7065 |
| **Minimum** | 0.699 | 0.699 | 0.699 | 0.301 | 0.000 | 0.301 |
| **Maximum** | 7.452 | 7.562 | 7.414 | 7.607 | 7.516 | 7.514 |
| **Quartile 1** | 2.709 | 2.738 | 2.803 | 2.621 | 2.620 | 2.682 |
| **Median** | 3.156 | 3.180 | 3.234 | 3.078 | 3.072 | 3.128 |
| **Quartile 3** | 3.582 | 3.609 | 3.656 | 3.515 | 3.504 | 3.561 |
| **Mean** | 3.135 | 3.170 | 3.221 | 3.060 | 3.053 | 3.112 |
| **Variance (n-1)** | 0.460 | 0.454 | 0.448 | 0.488 | 0.481 | 0.475 |
| **Standard deviation (n-1)** | 0.678 | 0.674 | 0.669 | 0.699 | 0.694 | 0.689 |
|  |  |  |  |  |  |  |
| **Expression Statistics**** | **STM 2683** | | | **STM 4661** | | |
|  | **Zinc /Control** | **Cadmium /Control** | **Cadmium /Zinc** | **Zinc /Control** | **Cadmium /Control** | **Cadmium /Zinc** |
| **Nb. of observations** | 6860 | 6860 | 6860 | 7013 | 7013 | 7013 |
| **Minimum** | -4.370 | -1.750 | -1.470 | -2.980 | -1.440 | -1.950 |
| **Maximum** | 4.650 | 3.480 | 6.680 | 5.320 | 4.190 | 7.150 |
| **Quartile 1** | -0.130 | -0.140 | -0.130 | -0.140 | -0.110 | -0.110 |
| **Median** | 0.000 | 0.000 | 0.000 | 0.000 | 0.000 | 0.000 |
| **Quartile 3** | 0.130 | 0.130 | 0.120 | 0.130 | 0.120 | 0.110 |
| **Mean** | 0.000 | -0.003 | -0.006 | 0.002 | 0.011 | 0.001 |
| **Variance (n-1)** | 0.069 | 0.064 | 0.075 | 0.069 | 0.058 | 0.068 |
| **Standard deviation (n-1)** | 0.262 | 0.252 | 0.274 | 0.262 | 0.240 | 0.261 |

* Descriptive statistics of read counts obtained for the two isolates (STM 2683 or STM 4661) and each treatment (Cadmium, Control and Zinc) obtained from all genomic objects (Nb. of observations) after logarithmic transformation

** Descriptive statistics of differential expression obtained for the two isolates and all possible comparisons (Zinc / Control ; Cadmium / Control ; Cadmium / Zinc) obtained from all genomic objects excluding tRNAs and rRNAs (Nb. of observations) after logarithmic base-2 transformation
