# Supplementary material for: Genome-wide transcriptional responses of two metal-tolerant symbiotic Mesorhizobium isolates to Zinc and Cadmium exposure
Source: BMC Genomics. 2013 Apr 30;14:292. doi: 10.1186/1471-2164-14-292 (PMC3668242; doi:10.1186/1471-2164-14-292)
Supplement: Additional file 3 — Histogram representing the log10-transformed read count number per CDS for all treatments. [file 1471-2164-14-292-S3.pptx]

## Slide 1
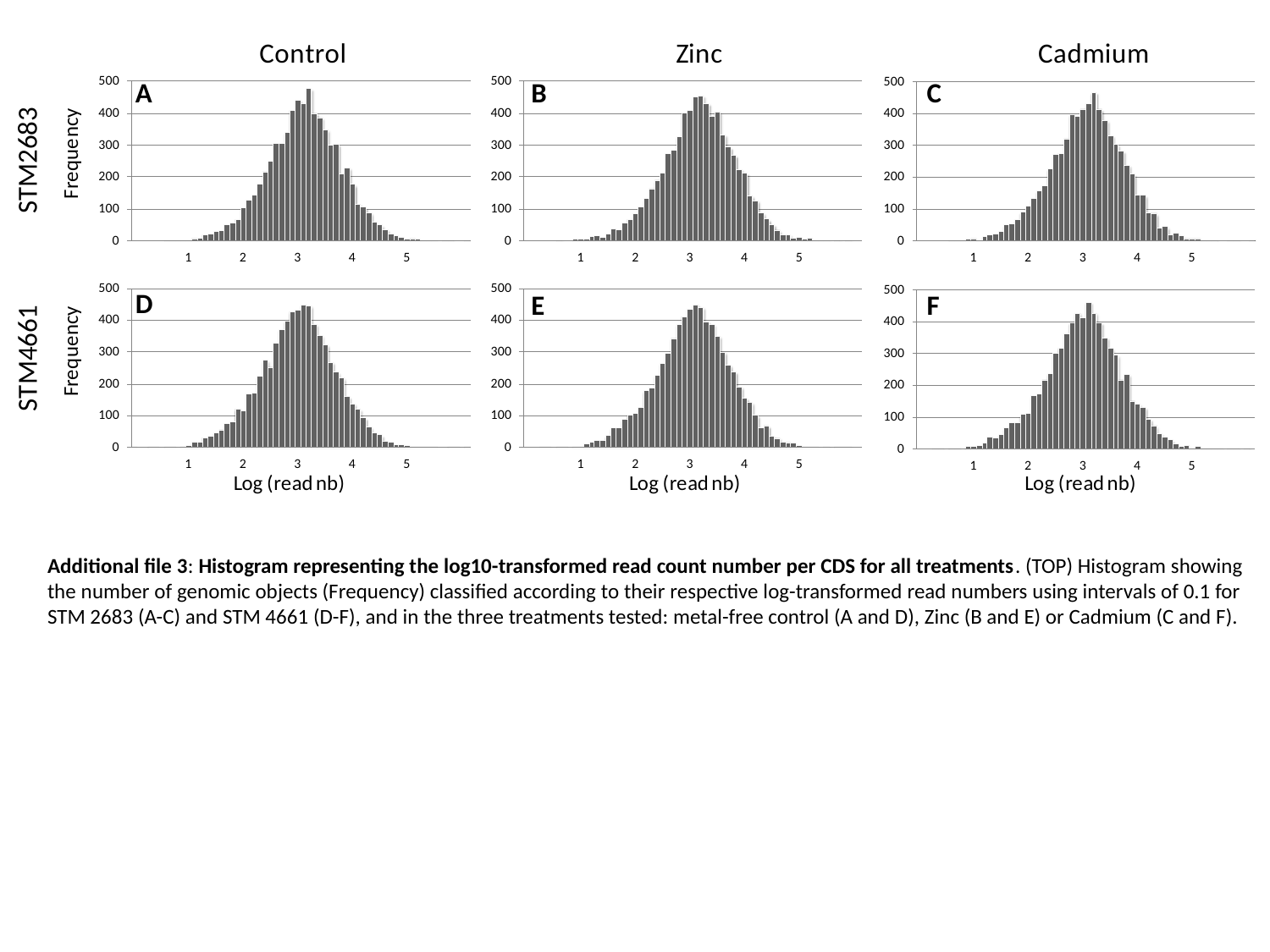

Additional file 3: Histogram representing the log10-transformed read count number per CDS for all treatments. (TOP) Histogram showing the number of genomic objects (Frequency) classified according to their respective log-transformed read numbers using intervals of 0.1 for STM 2683 (A-C) and STM 4661 (D-F), and in the three treatments tested: metal-free control (A and D), Zinc (B and E) or Cadmium (C and F).
