# Supplementary material for: Genome-wide transcriptional responses of two metal-tolerant symbiotic Mesorhizobium isolates to Zinc and Cadmium exposure
Source: BMC Genomics. 2013 Apr 30;14:292. doi: 10.1186/1471-2164-14-292 (PMC3668242; doi:10.1186/1471-2164-14-292)
Supplement: Additional file 4 — Histogram representing the log2-fold changes obtained for all CDS and all comparisons. [file 1471-2164-14-292-S4.pptx]

## Slide 1
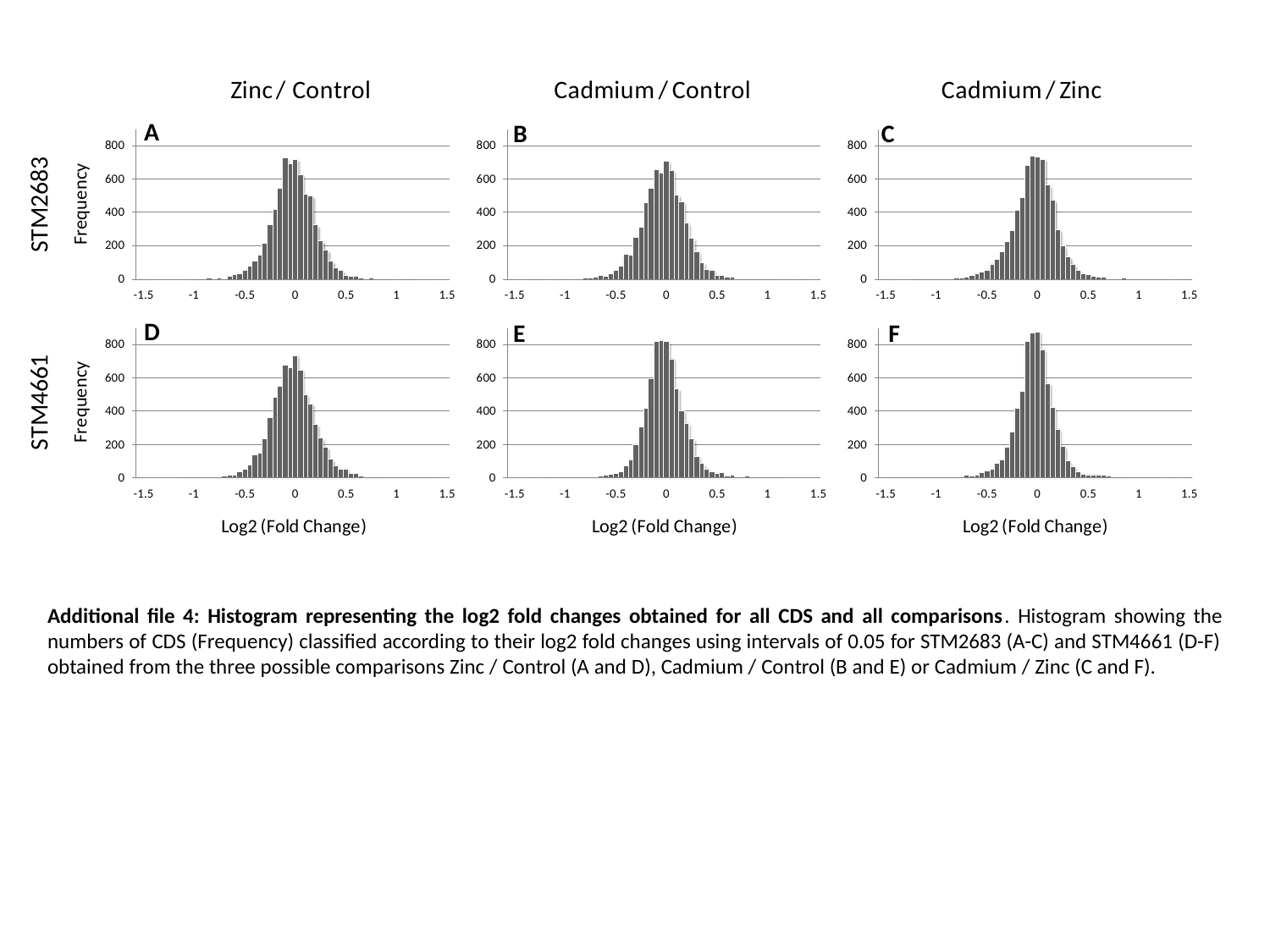

Additional file 4: Histogram representing the log2 fold changes obtained for all CDS and all comparisons. Histogram showing the numbers of CDS (Frequency) classified according to their log2 fold changes using intervals of 0.05 for STM2683 (A-C) and STM4661 (D-F) obtained from the three possible comparisons Zinc / Control (A and D), Cadmium / Control (B and E) or Cadmium / Zinc (C and F).
