# Supplementary material for: Genome-wide transcriptional responses of two metal-tolerant symbiotic Mesorhizobium isolates to Zinc and Cadmium exposure
Source: BMC Genomics. 2013 Apr 30;14:292. doi: 10.1186/1471-2164-14-292 (PMC3668242; doi:10.1186/1471-2164-14-292)
Supplement: Additional file 5 — Scatter plot representations of RNAseq data. [file 1471-2164-14-292-S5.pptx]

## Slide 1
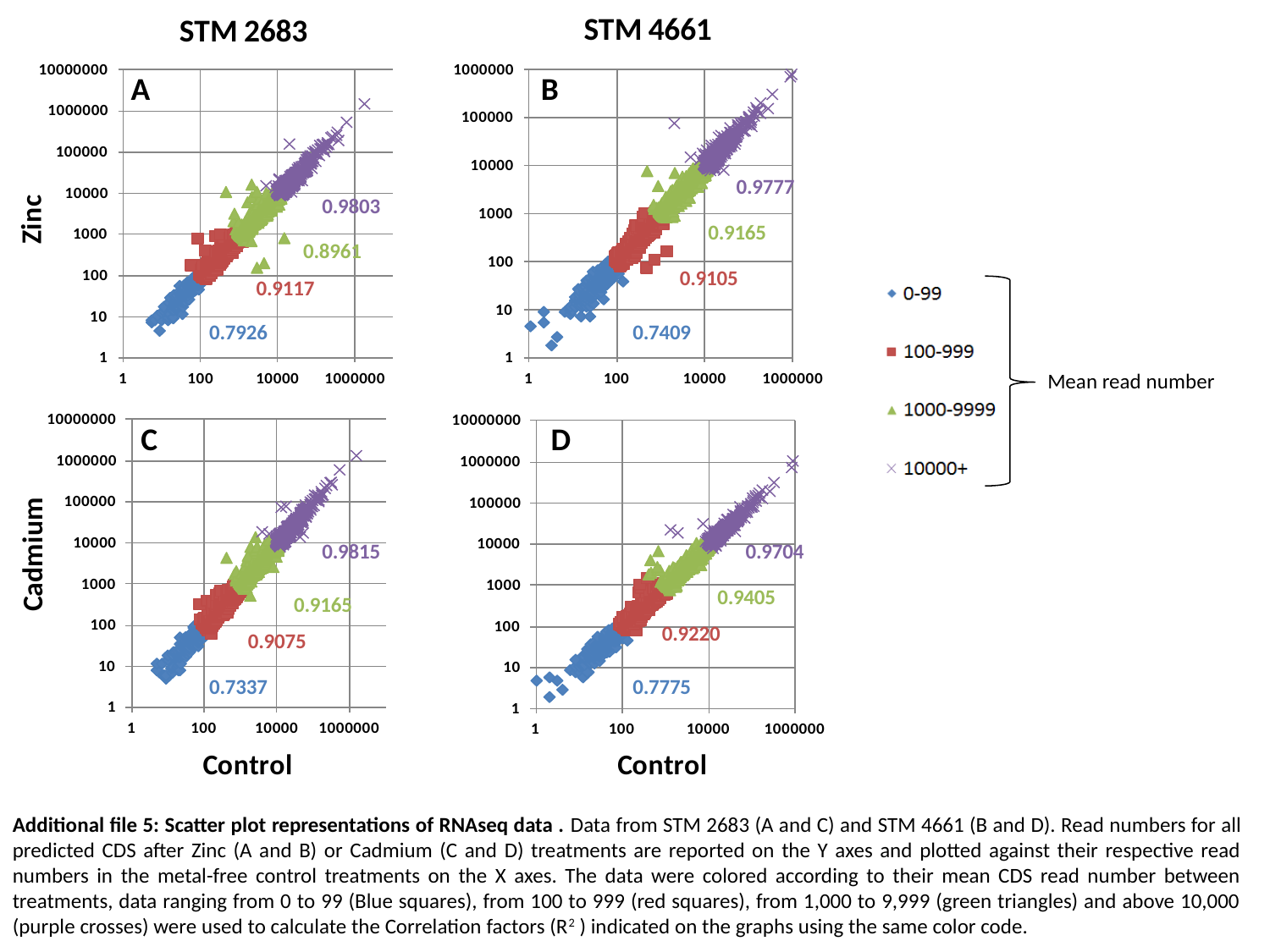

Mean read number
Additional file 5: Scatter plot representations of RNAseq data . Data from STM 2683 (A and C) and STM 4661 (B and D). Read numbers for all predicted CDS after Zinc (A and B) or Cadmium (C and D) treatments are reported on the Y axes and plotted against their respective read numbers in the metal-free control treatments on the X axes. The data were colored according to their mean CDS read number between treatments, data ranging from 0 to 99 (Blue squares), from 100 to 999 (red squares), from 1,000 to 9,999 (green triangles) and above 10,000 (purple crosses) were used to calculate the Correlation factors (R2 ) indicated on the graphs using the same color code.
