# Supplementary material for: Genome-wide transcriptional responses of two metal-tolerant symbiotic Mesorhizobium isolates to Zinc and Cadmium exposure
Source: BMC Genomics. 2013 Apr 30;14:292. doi: 10.1186/1471-2164-14-292 (PMC3668242; doi:10.1186/1471-2164-14-292)
Supplement: Additional file 6 — MA plot representations of RNAseq data. [file 1471-2164-14-292-S6.pptx]

## Slide 1
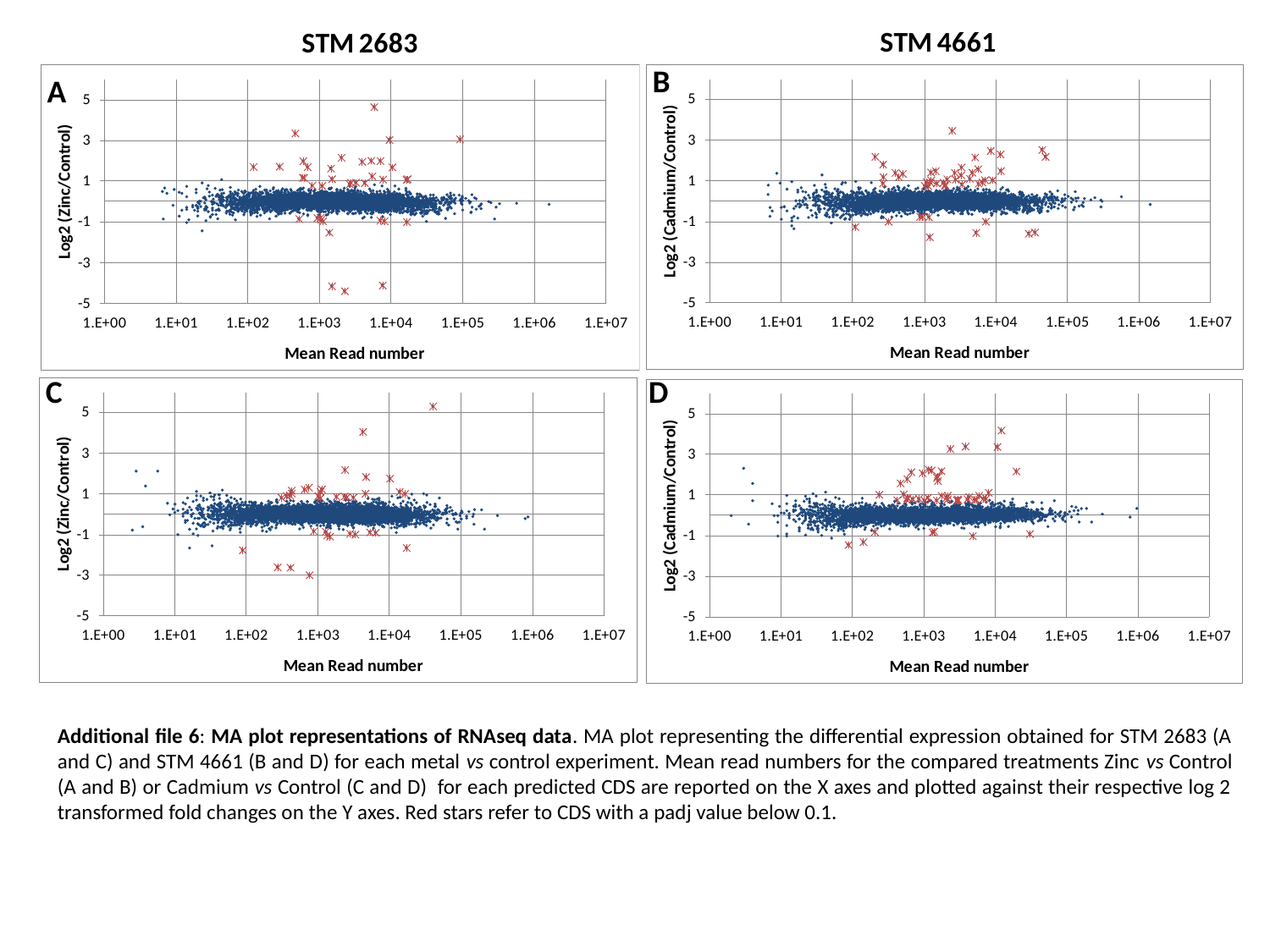

Additional file 6: MA plot representations of RNAseq data. MA plot representing the differential expression obtained for STM 2683 (A and C) and STM 4661 (B and D) for each metal vs control experiment. Mean read numbers for the compared treatments Zinc vs Control (A and B) or Cadmium vs Control (C and D) for each predicted CDS are reported on the X axes and plotted against their respective log 2 transformed fold changes on the Y axes. Red stars refer to CDS with a padj value below 0.1.
