# Supplementary material for: Genome-wide transcriptional responses of two metal-tolerant symbiotic Mesorhizobium isolates to Zinc and Cadmium exposure
Source: BMC Genomics. 2013 Apr 30;14:292. doi: 10.1186/1471-2164-14-292 (PMC3668242; doi:10.1186/1471-2164-14-292)
Supplement: Additional file 7 — Comparison between quantitative PCR and RNAseq data. [file 1471-2164-14-292-S7.pptx]

## Slide 1
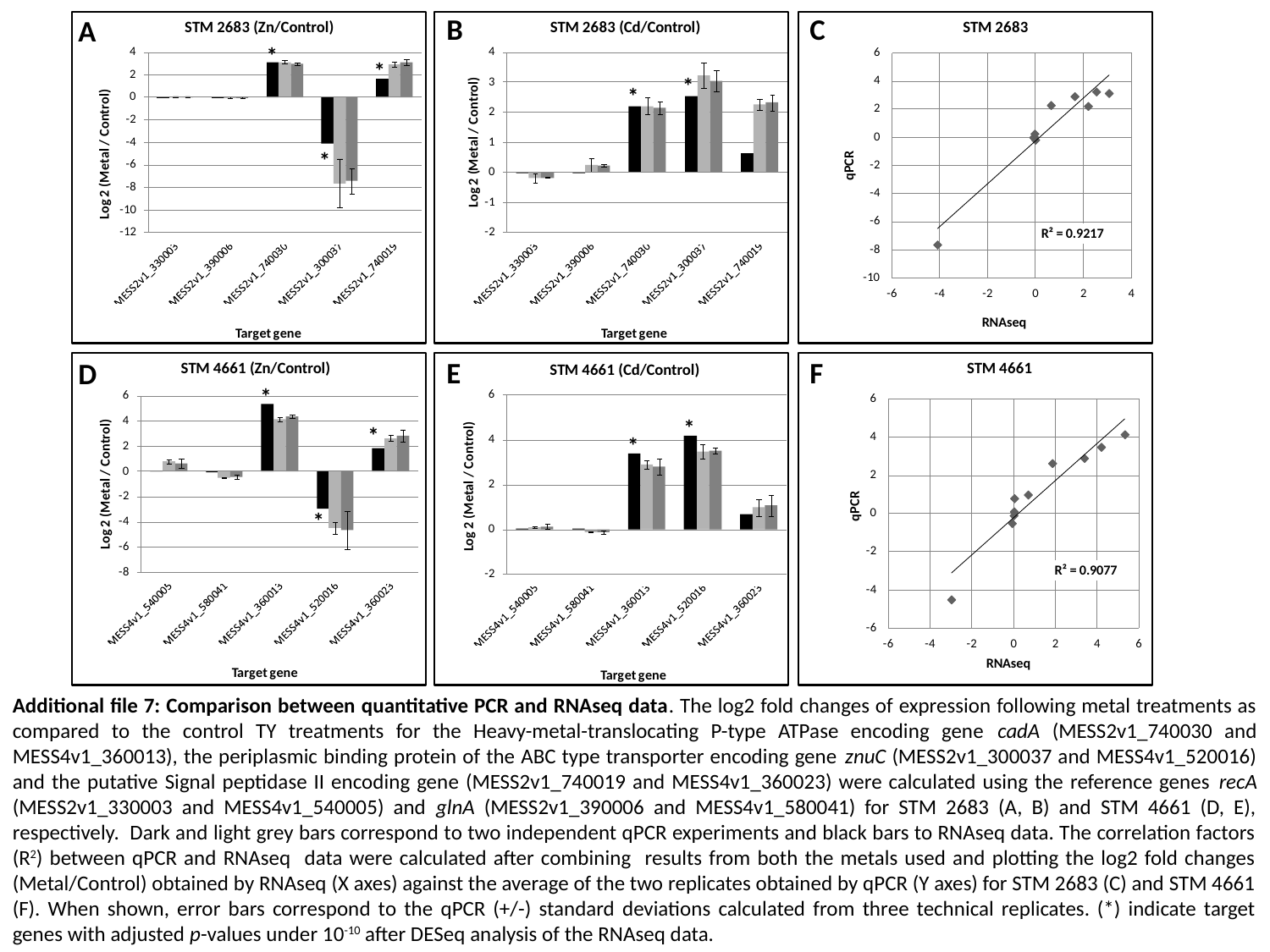

Additional file 7: Comparison between quantitative PCR and RNAseq data. The log2 fold changes of expression following metal treatments as compared to the control TY treatments for the Heavy-metal-translocating P-type ATPase encoding gene cadA (MESS2v1_740030 and MESS4v1_360013), the periplasmic binding protein of the ABC type transporter encoding gene znuC (MESS2v1_300037 and MESS4v1_520016) and the putative Signal peptidase II encoding gene (MESS2v1_740019 and MESS4v1_360023) were calculated using the reference genes recA (MESS2v1_330003 and MESS4v1_540005) and glnA (MESS2v1_390006 and MESS4v1_580041) for STM 2683 (A, B) and STM 4661 (D, E), respectively. Dark and light grey bars correspond to two independent qPCR experiments and black bars to RNAseq data. The correlation factors (R2) between qPCR and RNAseq data were calculated after combining results from both the metals used and plotting the log2 fold changes (Metal/Control) obtained by RNAseq (X axes) against the average of the two replicates obtained by qPCR (Y axes) for STM 2683 (C) and STM 4661 (F). When shown, error bars correspond to the qPCR (+/-) standard deviations calculated from three technical replicates. (*) indicate target genes with adjusted p-values under 10-10 after DESeq analysis of the RNAseq data.
