# Supplementary material for: Genome-wide transcriptional responses of two metal-tolerant symbiotic Mesorhizobium isolates to Zinc and Cadmium exposure
Source: BMC Genomics. 2013 Apr 30;14:292. doi: 10.1186/1471-2164-14-292 (PMC3668242; doi:10.1186/1471-2164-14-292)
Supplement: Additional file 8 — Estimation of the biological variations. [file 1471-2164-14-292-S8.pptx]

## Slide 1
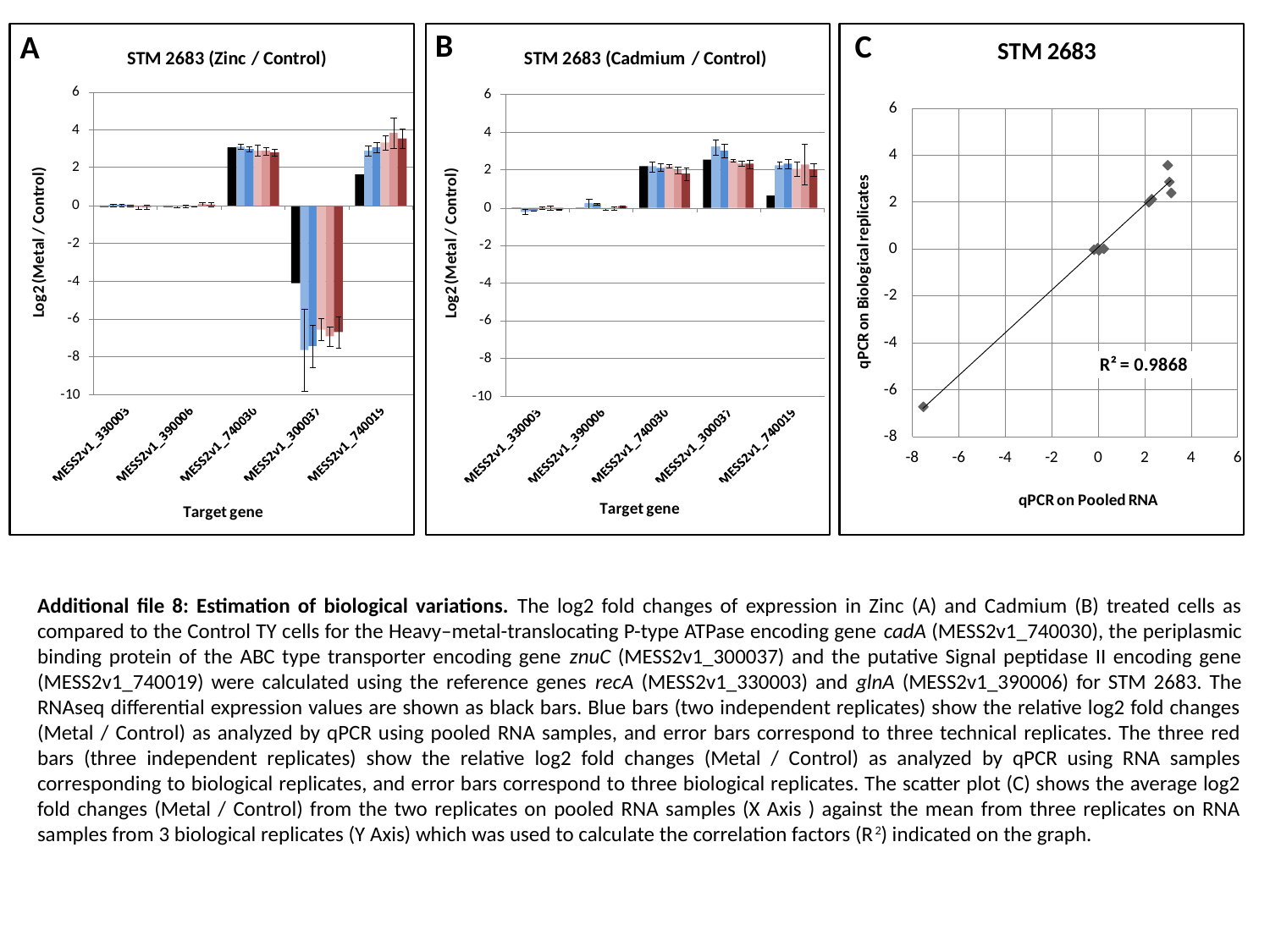

Additional file 8: Estimation of biological variations. The log2 fold changes of expression in Zinc (A) and Cadmium (B) treated cells as compared to the Control TY cells for the Heavy–metal-translocating P-type ATPase encoding gene cadA (MESS2v1_740030), the periplasmic binding protein of the ABC type transporter encoding gene znuC (MESS2v1_300037) and the putative Signal peptidase II encoding gene (MESS2v1_740019) were calculated using the reference genes recA (MESS2v1_330003) and glnA (MESS2v1_390006) for STM 2683. The RNAseq differential expression values are shown as black bars. Blue bars (two independent replicates) show the relative log2 fold changes (Metal / Control) as analyzed by qPCR using pooled RNA samples, and error bars correspond to three technical replicates. The three red bars (three independent replicates) show the relative log2 fold changes (Metal / Control) as analyzed by qPCR using RNA samples corresponding to biological replicates, and error bars correspond to three biological replicates. The scatter plot (C) shows the average log2 fold changes (Metal / Control) from the two replicates on pooled RNA samples (X Axis ) against the mean from three replicates on RNA samples from 3 biological replicates (Y Axis) which was used to calculate the correlation factors (R2) indicated on the graph.
