# Supplementary material for: Genome-wide transcriptional responses of two metal-tolerant symbiotic Mesorhizobium isolates to Zinc and Cadmium exposure
Source: BMC Genomics. 2013 Apr 30;14:292. doi: 10.1186/1471-2164-14-292 (PMC3668242; doi:10.1186/1471-2164-14-292)
Supplement: Additional file 9 — Global effect of the metal treatment on the regulation of genes as classified by COG functional categories. [file 1471-2164-14-292-S9.pptx]

## Slide 1
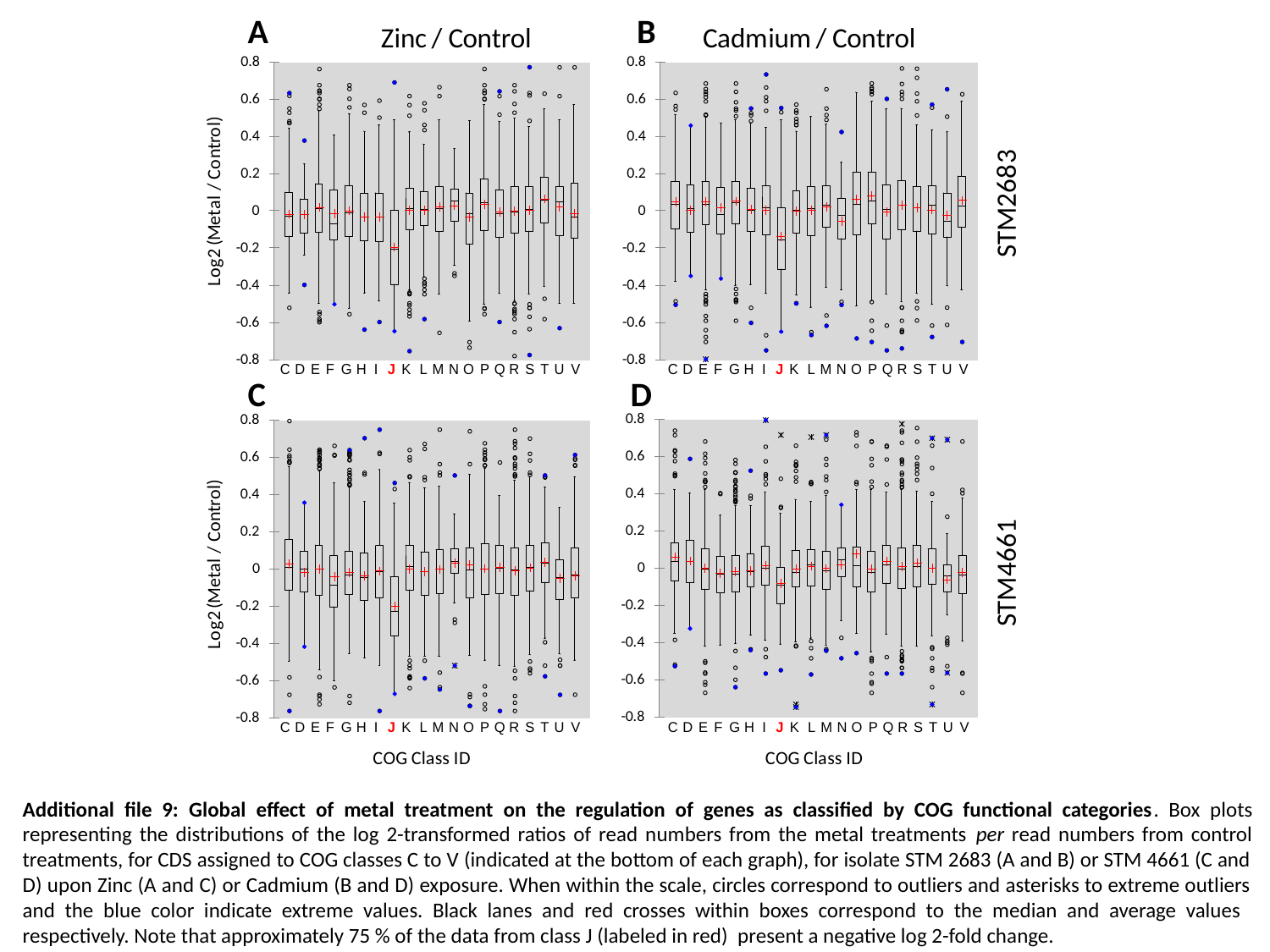

Additional file 9: Global effect of metal treatment on the regulation of genes as classified by COG functional categories. Box plots representing the distributions of the log 2-transformed ratios of read numbers from the metal treatments per read numbers from control treatments, for CDS assigned to COG classes C to V (indicated at the bottom of each graph), for isolate STM 2683 (A and B) or STM 4661 (C and D) upon Zinc (A and C) or Cadmium (B and D) exposure. When within the scale, circles correspond to outliers and asterisks to extreme outliers and the blue color indicate extreme values. Black lanes and red crosses within boxes correspond to the median and average values respectively. Note that approximately 75 % of the data from class J (labeled in red) present a negative log 2-fold change.
