# Supplementary material for: Genome-wide transcriptional responses of two metal-tolerant symbiotic Mesorhizobium isolates to Zinc and Cadmium exposure
Source: BMC Genomics. 2013 Apr 30;14:292. doi: 10.1186/1471-2164-14-292 (PMC3668242; doi:10.1186/1471-2164-14-292)
Supplement: Additional file 10 — Strains, plasmids and primers used [59],[60]. [file 1471-2164-14-292-S10.docx]

**Additional file 10**: Strains, plasmids and primers used.

| **Strains**  **Plasmids**  **Primers** | **Characteristics** | **Source/ reference / amplified region** |
| --- | --- | --- |
| **Strains** |  |  |
| ***Escherichia coli* strains** |  |  |
| DH10B | F- *endA1 recA1 galE15 galK16 nupG rpsL* Δ*lac*X74 Φ80l*ac*ZΔM15 *araD139* Δ(ara,leu)7697 | [59] |
|  | *mcrA* Δ(*mrr-hsdRMS-mcrBC*) λ- |  |
| XL2 Blue Ultra-competent Cells | endA1 supE44 thi-1 hsdR17 recA1 gyrA96 relA1 lac [F´ proAB lacIqZΔM15 Tn10 (Tetr) Amy Camr] | Stratagene |
|  |  |  |
| ***Mesorhizobium* strains** |  |  |
| *M. metallidurans* STM 2683^T^ | wild-type from the Avinieres mine | [7] |
| *Mesorhizobium* sp. STM 4661 | wild-type from the Eylie mine | This study |
| *M. tianshanense* ORS 2740^T^ | wild-type | [60] |
| *Mesorhizobium* sp. STM 2682 | wild-type from the Prunarede site | [5] |
|  |  |  |
| **Plasmids** |  |  |
| pGEM-T Easy | lacPOZ, cloning vector | Promega |
| pGEM-*cad*Ap | pGEM-T Easy containing the HindIII *cad*A promotor, Ap^R^ | Maynaud *et al.* (unpublished observation) |
| pGEMt-*znu*Cp | pGEM-T Easy containing the HindIII *znu*C promotor, Ap^R^ | This study |
| pPROBE-GT | promoter-probe vector with gfp gene, Gm^R^ | [49] |
| pGT-*cad*Ap | pPROBE GT containing HindIII *cad*A promotor downstream *gfp* gene, Gm^R^ | Maynaud *et al.* (unpublished observation) |
| pGT-*znu*Cp | pPROBE GT containing HindIII *znu*C promotor downstream *gfp* gene, Gm^R^ | This study |
|  |  |  |
| **Primers** |  |  |
| STM 2953-Forward | 5’-AAGCTTAGAGTCGCGGTTCGC-3’ | *This study / znu*C promoter region |
| STM 2954-Reverse | 5’-AAGCTTCCAGCGTCAGGTCG-3’ | *This study / znu*C promoter region |
|  |  |  |
