# Supplementary material for: Genome-wide transcriptional responses of two metal-tolerant symbiotic Mesorhizobium isolates to Zinc and Cadmium exposure
Source: BMC Genomics. 2013 Apr 30;14:292. doi: 10.1186/1471-2164-14-292 (PMC3668242; doi:10.1186/1471-2164-14-292)
Supplement: Additional file 11 — Primers used for quantitative PCR assays. [file 1471-2164-14-292-S11.docx]

**Additional file 11**: Primers used for quantitative PCR assays.

| **Primer name** | **Primer sequence (5' - 3')** | **STM 2683 CDS** | **STM 4661 CDS** | **Product (gene)** |
| --- | --- | --- | --- | --- |
| STM 2772 Forward | CGTCCCGCCGCTCGTCTTTG | MESS2v1_740030 | MESS4v1_360013 | Heavy-metal-translocating P-type ATPase (*cad*A) |
| STM 2773-Reverse | CCCGCCCTTCATCAGCAATCCC |  |  |  |
| STM 2782-Forward | CAGCGGCAAGACGGTCAC | MESS2v1_300037 | MESS4v1_520016 | ABC transporter, periplasmic binding protein (*znu*A) |
| STM 2773-Reverse | CGCCTTCACTTCGCCTTCG |  |  |  |
| STM 2957-Forward | TGGTGCTCGGCTACAATGAAGG | MESS2v1_740019 | MESS4v1_360023 | Signal peptidase II, Aspartic peptidase, MEROPS family A08 |
| STM 2958-Reverse | CCACTCGCTCCGCCAGAAATG |  |  |  |
| STM 2778-Forward | CGGTGCTGCTCGCCTATTC | MESS2v1_390006 | MESS4v1_580041 | Glutamine synthetase I (*gln*A) |
| STM 2779-Reverse | TCCTTGTCCATCGGCTGTCC |  |  |  |
| STM 2776-Forward | GGCGAGGGCGTGTCCAAG | MESS2v1_330003 | MESS4v1_540005 | Recombinase A (*rec*A) |
| STM 2777-Reverse | TGCCGCAGTGCCAGTTCG |  |  |  |
